# Comparative physiological responses and transcriptome analysis reveal the roles of melatonin and serotonin in regulating growth and metabolism in *Arabidopsis*

**DOI:** 10.1186/s12870-018-1548-2

**Published:** 2018-12-18

**Authors:** Jinpeng Wan, Ping Zhang, Ruling Wang, Liangliang Sun, Qiong Ju, Jin Xu

**Affiliations:** 1 CAS Key Laboratory of Tropical Plant Resources and Sustainable Use, Xishuangbanna Tropical Botanical Garden, Chinese Academy of Sciences, Menglun, Mengla, 666303 Yunnan China; 2 University of Chinese Academy of Sciences, Beijing, 100049 China

**Keywords:** Melatonin, Serotonin, Root system development, Gene expression reprogramming, Metabolic adjustment

## Abstract

**Background:**

Melatonin and serotonin are well-known signaling molecules that mediate multiple physiological activities in plants, including stress defense, growth, development, and morphogenesis, but their underlying mechanisms have not yet been thoroughly elucidated. In this study, we investigated the roles of melatonin and serotonin in modulating plant growth and defense by integrating physiological and transcriptome analyses in *Arabidopsis*.

**Results:**

Moderate concentrations of melatonin and serotonin did not affect primary root (PR) growth but markedly induced lateral root (LR) formation. Both melatonin and serotonin locally induced the expression of the cell-wall-remodeling-related genes *LBD16* and *XTR6*, thereby inducing LR development. Our data support the idea that melatonin and serotonin lack any auxin-like activity. Treatment with 50 μM serotonin significantly improved PSII activity, and the transcriptome data supported this result. Melatonin and serotonin slightly affected glycolysis and the TCA cycle; however, they markedly regulated the catabolism of several key amino acids, thereby affecting carbon metabolism and energy metabolism. Melatonin and serotonin improved iron (Fe) deficiency tolerance by inducing Fe-responsive gene expression.

**Conclusions:**

Overall, our results from the physiological and transcriptome analyses reveal the roles of melatonin and serotonin in modulating plant growth and stress responses and provide insight into novel crop production strategies using these two phytoneurotransmitters.

**Electronic supplementary material:**

The online version of this article (10.1186/s12870-018-1548-2) contains supplementary material, which is available to authorized users.

## Background

Melatonin (N-acetyl-5-methoxytryptamine) and its precursor serotonin (5-hydroxytryptamine) are two highly conserved molecules that mediate a series of physiological activities in humans, animals, and plants [1–5]. Under normal conditions, the melatonin content is relatively low; however, biotic and abiotic stresses, such as pathogen infection, low temperature, salt, drought, iron deficiency, and heavy metal toxicity, markedly induce melatonin accumulation in plants [6–12]. Previous studies have indicated that exogenous melatonin improves stress tolerance by both directly and indirectly regulating redox status and eliminating oxidative stress in plants [13, 14]. The melatonin biosynthesis-deficient mutant *snat* exhibits susceptibility to an avirulent pathogen [15], and exogenous melatonin improves pathogen resistance against *Pseudomonas syringae* in *Arabidopsis* [16]. Treatment with melatonin also improves resistance against Marssonina apple blotch in apple leaves [17], indicating that melatonin plays a role in the defense response in plants. A recent study showed that melatonin regulates carbohydrate metabolism during sugar starvation in plants, thereby improving stress tolerance [18]. However, the molecular mechanisms underlying melatonin-mediated defense and sugar metabolism have not been thoroughly elucidated to date.

The serotonin level is also notably low in young leaves; however, nutrient deficiency, pathogen infection, and senescence acutely induce the accumulation of serotonin in plants [1, 19, 20]. The elevated serotonin levels strengthen cell walls in infected tissues, improving pathogen tolerance [1, 20]. Senescent tissues accumulate high concentrations of serotonin, and serotonin shows senescence-slowing activity through its high antioxidant activity in leaves [1]. Exogenous serotonin suppresses the growth of fungal hyphae in rice leaves [21]. By contrast, suppression of serotonin biosynthesis improves the resistance of rice to planthoppers and stem borers, two types of destructive pests of rice, and supplementation with serotonin in an artificial diet enhances the performance of the two insect pests, indicating that serotonin also plays an important role in the defense response in plants.

Although serotonin is the precursor of melatonin, serotonin is more highly inducible by senescence, pathogens, and environmental stresses. Supplementation with exogenous serotonin or overexpression of *TDC* genes does not greatly induce melatonin accumulation in plants, suggesting the existence of pathways for the conversion of melatonin to serotonin [22, 23]. Recently, Lee et al. (2018) showed that serotonin *N*-acetyltransferase (SNAT) and *N*-acetylserotonin deacetylase (ASDAC) play roles in a reversible melatonin biosynthesis pathway [24]. SNAT promotes melatonin biosynthesis, whereas ASDAC restricts it, indicating that the melatonin content is tightly controlled by these two enzymes to maintain an optimal level in plants [24].

In addition to their roles in stress defense, both melatonin and serotonin modulate plant growth, development, and morphogenesis. Melatonin and serotonin improve shoot organogenesis from root cultures by regulating auxin signaling [25, 26]. Erland et al. (2017) found that melatonin and serotonin function in cooperation with their metabolites in a cascade of phytochemical responses involving multiple pathways and the phytohormone network, including cytokinin, abscisic acid (ABA), and salicylic acid (SA) signaling, to direct morphogenesis and protect photosynthesis in *Hypericum perforatum* explants in vitro [27]. The results of these studies indicate that melatonin and serotonin regulate plant growth and morphogenesis through the integration of phytohormone signaling pathways.

Identification of the response mechanisms of plants to melatonin and serotonin is important to help formulate strategies for crop improvement. In this study, we compared the physiological responses of *Arabidopsis* to exogenous melatonin and serotonin. We investigated the molecular mechanisms responsible for melatonin- and serotonin-mediated metabolism and pathogen resistance using a combination of transcriptome and physiological analyses. Our results provide a basis for further elucidating the molecular mechanisms of melatonin- and serotonin-mediated plant growth, development, and defense and provide insight into novel strategies for crop production using these two plant growth regulators.

## Methods

### Plant materials and growth conditions

*Arabidopsis* seeds were purchased from NASC (the European Arabidopsis Stock Centre). The seeds were sterilized and stratified at 4 °C for 2 d before sowing on vertically oriented agar medium containing one-fourth-strength MS medium (Sigma-Aldrich), pH 5.7, supplemented with 1% (m/v) agar and 1.5% (m/v) sucrose. For the iron (Fe) deficiency tolerance assay, we used full-strength MS medium without Fe to augment the Fe deficiency phenotype. The seedlings were grown in growth chambers at 22 °C under 16 h light period (120 μmol m^− 2^ s^− 1^). Five-d-old seedlings were transferred to fresh medium with various concentrations of chemicals, such as 10 μM or 50 μM serotonin or melatonin, and grown for an additional 2–4 d [2]. Serotonin and melatonin were purchased from TCI (Tokyo, Japan) and dissolved in dimethyl sulfoxide (DMSO) to prepare stock solutions.

### Determination of primary root length, lateral root initiation events, lateral root primordium initiation, and lateral root numbers

After the seedlings were treated with different concentrations of chemicals, photographs were scanned using an Epson Perfection V500 Photo scanner (Japan), and the primary root (PR) length and lateral root (LR) numbers were determined using ImageJ software (version 1.51j8). After 5-day-old Col-0 seedlings were transferred to plates supplemented with chemicals for an additional 4 days, the number of LR primordia (LRPs) was quantified as described by Zhang et al. (1999) [28]. The development of each LR or LRP was classified into four stages: from initiation to up to 3 cell layers (stage I); more than 3 cell layers without emergence (stage II); emerged, but with a length of less than 0.5 mm (stage III); and a length of 0.5 mm or more (stage IV). Only stage IV was considered to characterize LRs.

### Chlorophyll fluorescence measurements

Chlorophyll fluorescence parameters were measured using an LI-6800 system (LI-COR, America) following the manufacturer’s instructions. After leaves were exposed to adequate dark adaptation, F_0_ (minimum fluorescence) and Fm (maximum fluorescence yield) were measured under weak light conditions. Then, the dark-adapted plants were exposed to a light intensity of 120 μmol m^− 2^ s^− 1^ for adequate light adaptation. When performing measurements of induction kinetics, the activated light intensity was set to 90 μmol m^− 2^ s^− 1^. The following parameters were recorded: F_0_’ (minimum fluorescence yield of light-adapted leaves), Fm′ (maximum fluorescence yield of light-adapted leaves), Fs (fluorescence in a stable state), and ΦPSII (effective quantum yield of PSII). The optimal photochemical efficiency of PSII in the dark (Fv/Fm) was calculated as (Fm-Fo)/Fm. The nonphotochemical quenching parameter (NPQ) was calculated as (Fm-Fm′)/Fm′, and the plastoquinone (PQ) redox state of PSII (1-qL) was calculated as (Fo’/Fs)(Fm′-Fs)/(Fm′-Fo’) [29].

### qRT-PCR analysis

The RNAiso Plus Kit (TaKaRa) was used to isolate total RNA from the samples subjected to the different treatments according to the manufacturer’s instructions. Then, the PrimeScript RT Reagent Kit with gDNA Eraser (TaKaRa) was used to perform reverse transcription. The UltraSYBR mixture (CWBIO) was used to perform quantitative reverse transcription (qRT)-PCR. *ACTIN2* (AT3G18780) served as an internal control [30, 31]. The cDNA produced was diluted 1:10, and 2 μL of diluted cDNA was used for qRT-PCR in a 7500 Real Time System (Applied Biosystems). The PCR cycling conditions were as follows: 95 °C for 10 min; 45 cycles at 95 °C for 10 s, 60 °C for 20 s, and 72 °C for 20 s; and 72 °C for 10 min. The specific primers that were used were as follows: *XTR6* forward 5’-CCTAAGCTCAAAGCCCACCA-3′ and reverse 5’-TGGAATCCTGAGCCTGAAGC-3′; and *LBD16* forward 5′- TGACCCTGTTTATGGATGTGTC-3′ and reverse 5’-TGATTGCAAGAAAGCCACCT-3′; *bHLH38* forward 5’-TGTTTCTTTAGTCTTTCATCCGCA-3′ and reverse 5’-CTCCGGCACCGTAATAGCTT-3′; *bHLH39* forward 5’-ATATCCTCAACAACGGCGGG-3′ and reverse 5’-AGGCAGGAAGACATGAACGG-3′; *bHLH100* forward 5’-ACGAGAAATGTGGACACTCGT-3′ and reverse 5’-CAACCCATGTTCGCGTTGTT-3′; *bHLH101* forward 5’-CTCTCAACGGATCGCAGCA-3′ and reverse 5’-TGGCGTAATCCCAAGAGCA-3′; *IRT1* forward 5’-TTCACTCGGTGGTCATTGGA-3′ and reverse 5’-CCGAATGGTGTTGTTACCGC-3′. For each primer pair, the DNA melting curve showed only one peak, and three biological replicates with three technical repetitions of qRT-PCR analysis were performed for each gene.

### GUS staining

The following transgenic lines *pCYCLINB1;1:CYCLINB1;1-GUS, pCYCLINB3;1:CYCLINB3;1-GUS, pCYCLINA3;1:CYCLINA3;1-GUS, pWOX5:GUS, QC25-GUS, pXTR6:GUS*, and *pLBD16:GUS* were used for histochemical GUS staining analysis [2, 30, 32, 33]. Treated seedlings were incubated in GUS staining solution containing 1 mM 5-bromo-4-chloro-3-indolyl-b-D-GlcA cyclohexyl-ammonium (Sigma-Aldrich), 0.5 mM potassium ferrocyanide, 0.5 mM potassium ferricyanide, and 10 mM EDTA in 50 mM sodium phosphate buffer (pH 7.0) [34]. The duration of GUS staining was as follows: 1 h for *pCYCLINA3;1:CYCLINA3;1-GUS*, *pCYCLINB3;1:CYCLINB3;1-GUS*, and *pLBD16:GUS*, 2 h for *pWOX5:GUS*, 2.5 h for *QC25-GUS*, 3 h for *pXTR6:GUS* and 3.5 h for *pCYCLINB1;1:CYCLINB1;1-GUS.* The samples were washed and placed in 75% (*w*/*v*) ethanol, and photographs were taken using a Carl Zeiss imaging system.

### GFP/YFP fluorescence microscopy

GFP and YFP fluorescence in the roots of the *DII-VENUS*, *AUX1-YFP*, *PIN1-GFP*, *PIN2-GFP*, *PIN4-GFP*, and *PIN7-GFP* marker lines was detected. After the seedlings were exposed to different chemicals for the indicated times, GFP and YFP were examined using a confocal laser scanning microscope (LSM710) following the manufacturer’s instructions (excitation wavelength of 488 nm and emission wavelength of 509 nm for GFP; excitation wavelength of 514 nm and emission wavelength of 527 nm for YFP).

### Quantification of free amino acids

Frozen tissue (500 mg) was ground in 1 mL of 20 mM cold HCl. Norleucine was used as an internal standard for measuring recovery. After derivatization, the mixture was transferred into a 100 μL glass insert in an amber vial and analyzed by HPLC (Rigol L3000-system, Rigol, Beijing, China) as described by Zhang et al. (2010) [35].

### Perl’s staining

For the in vivo localization of Fe in seedlings, we used Perl’s staining method according to Roschzttardtz et al. (2009) [36]. Briefly, seedlings were submerged in Perl’s staining solution (equal volumes of 4% (*v*/v) HCl and 4% (*w*/*v*) K-ferrocyanide) for 45 min [37]. DAB intensification was then performed as described by Meguro et al. (2007) [38], and photographs were taken using a Carl Zeiss imaging system.

### Statistical analysis

Each experiment was repeated at least three times with similar results. No less than 30 seedlings were analyzed for each treatment. The results are presented as the means ± SE. Student’s *t* test was used to determine the significance of the differences (*P* < 0.05).

## Results

### Root physiology in response to melatonin and serotonin

**Fig. 1:**
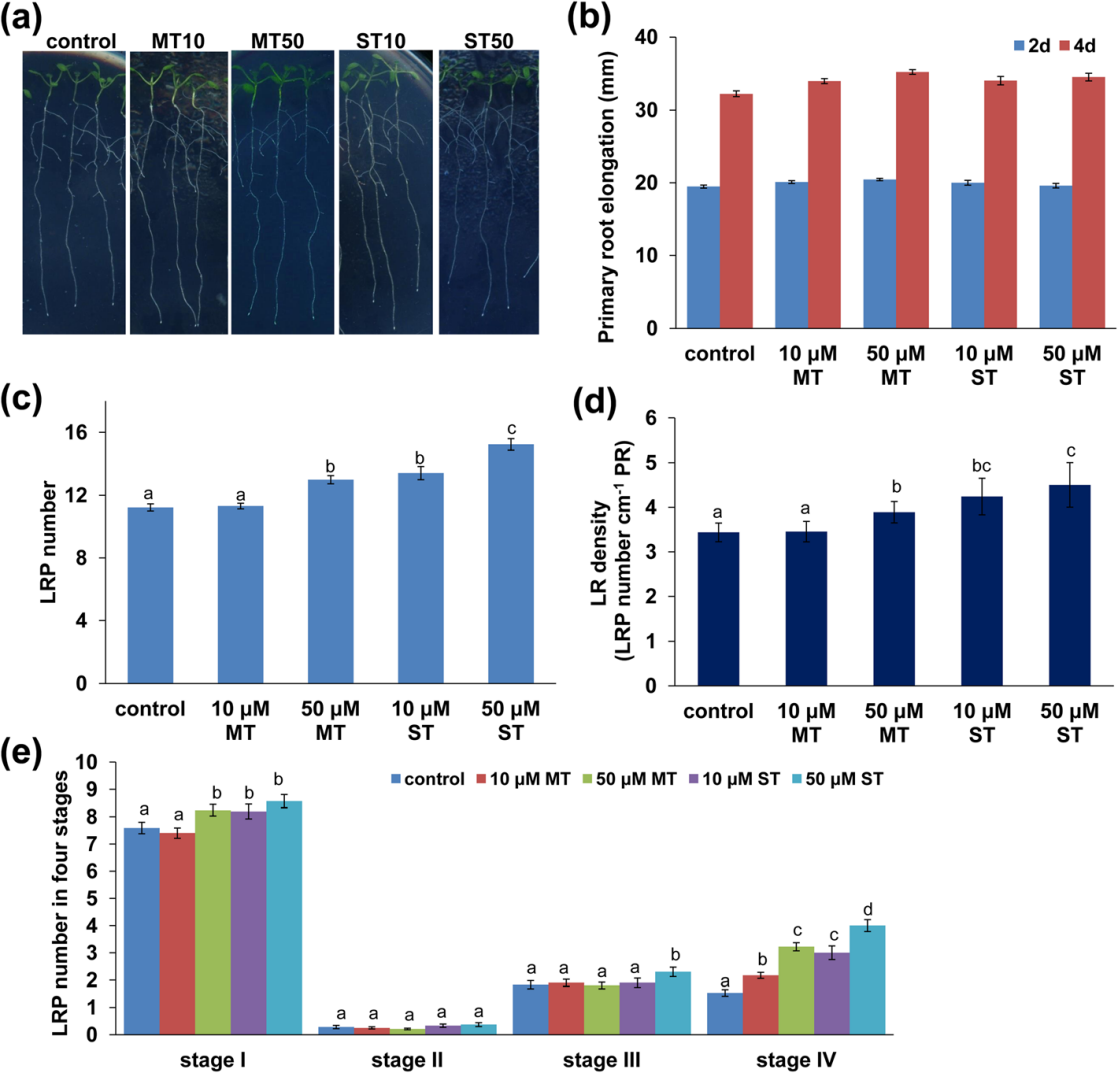
**a-e** Melatonin and serotonin regulate RSA. **a** Five-day-old seedlings were transferred to 1/4 MS medium containing 10 or 50 μM melatonin or serotonin for 4 days. **b** PR elongation, **c** LRP numbers, **d** LR density, and **e** LRP numbers at were determined four developmental stages, as indicated. MT, melatonin; ST, serotonin. Error bars represent SE. Different letters indicate significantly different values (*P* < 0.05 by Tukey’s test)

Previous studies have shown that moderate concentrations of melatonin and serotonin markedly induce lateral root (LR) formation, while high concentrations of melatonin (> 100 μM) and serotonin (> 300 μM) inhibit primary root (PR) growth [39, 40]. At an elevated concentration, serotonin also markedly inhibits LR development [39]. These studies further showed that high concentrations of melatonin and serotonin inhibit auxin transport and accumulation in roots, thereby reducing PR growth [39, 40]. To further explore the molecular mechanisms underlying melatonin- and serotonin-mediated plant growth and development, we first investigated root system growth in response to melatonin and serotonin. Five-day-old Col-0 seedlings were transferred to fresh 1/4 MS medium containing melatonin or serotonin. Supplementation with 10 or 50 μM melatonin or serotonin slightly but nonsignificantly increased PR growth (Fig. 1a, Additional file 1: Figure S1). We then analyzed the effects of melatonin and serotonin on the root meristematic cell division potential by using the *pCYCLINA3;1:CYCLINA3;1-GUS*, *pCYCLINB1;1:CYCLINB1;1-GUS*, and *pCYCLINB1;1:CYCLINB3;1-GUS* transgenic cell cycle marker lines and assessed stem cell niche activity by using the *QC25-GUS* and *pWOX5-GUS* transgenic lines [32]. GUS staining of all three cell cycle marker lines (Additional file 2: Figure S2a) and two stem cell marker lines (Additional file 2: Figure S2b) was unaffected by exogenous melatonin and serotonin. These results indicated that moderate concentrations of melatonin and serotonin did not affect root meristematic cell division and stem cell niche activity and therefore did not affect PR growth.

Although moderate concentrations of melatonin and serotonin did not affect PR growth, they did induce LR formation (Fig. 1b and c). The number of LR initiation events increased by 16% in the presence of 50 μM melatonin and by 19.5 and 36% in the presence of 10 and 50 μM serotonin, respectively (Fig. 1b). To further explore the effects of melatonin and serotonin on LR development, we analyzed LR primordium (LRP) initiation and found that it was significantly increased in stage I and stage IV after exposure to melatonin or serotonin (Fig. 1d).

**Fig. 2:**
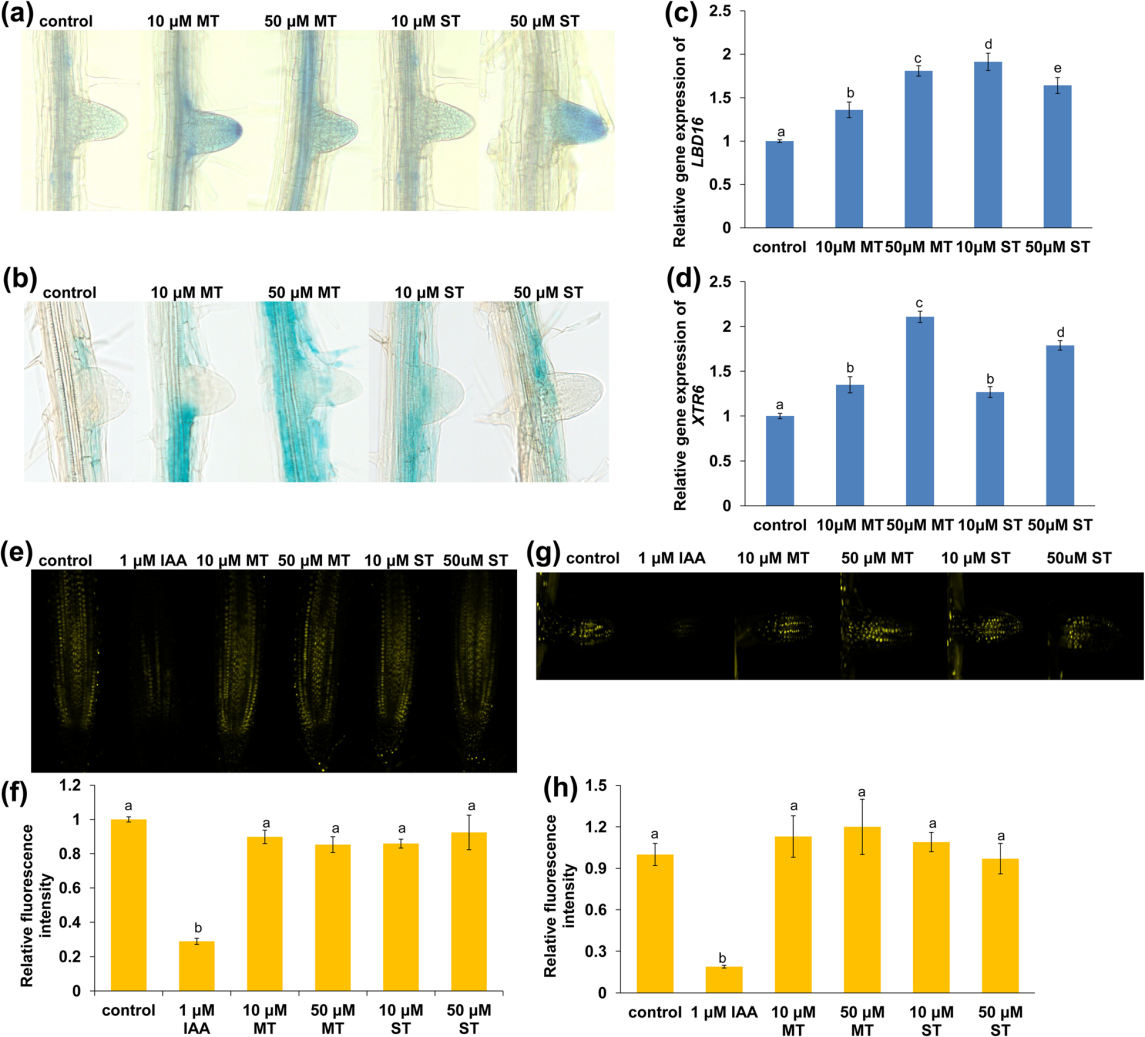
**a-g** *LBD16* and *XTR6* are involved in melatonin- or serotonin-mediated LR development. **a**, **b** Five-day-old *proLBD16:GUS* (**a**) and *proXTR6:GUS* (**b**) seedlings were transferred to 1/4 MS medium containing 10 or 50 μM melatonin or serotonin for 4 days. **c**, **d** qRT-PCR analysis of *LBD16* (**c**) and *XTR6* (**d**) gene expression in the roots of Col-0 seedlings treated with 10 or 50 μM melatonin or serotonin for 2 days. The expression levels of the indicated genes in untreated roots were set to 1. **e-h** Effects of melatonin and serotonin on auxin accumulation in roots. **e** YFP fluorescence in the PR tips of 5-day-old *DII-VENUS* seedlings exposed to 1 μM IAA or 10 or 50 μM melatonin or serotonin for 4 days and (**f**) quantification of DII-VENUS fluorescence intensity in plants treated as in (**e**). **g** YFP fluorescence in the LR tips of 5-day-old *DII-VENUS* seedlings exposed to 1 μM IAA or 10 or 50 μM melatonin or serotonin for 4 days and (**h**) quantification of the DII-VENUS fluorescence intensity in plants treated as in (**g**). The fluorescence intensity in untreated roots was set to 1. MT, melatonin; ST, serotonin. Error bars represent the SE. Different letters indicate significantly different values (*P* < 0.05 by Tukey’s test)

*LBD16*, an LR marker gene, plays an important role in modulating LR initiation and LRP development [41]. We therefore examined *LBD16* expression in melatonin- and serotonin-treated roots using *pLBD16:GUS* transgenic lines. Histochemical staining showed that GUS activity was higher in melatonin- and serotonin-treated root steles and LRs (Fig. 2a). The *XTR6* gene encodes a xyloglucan:xyloglucosyl transferase that controls cell wall remodeling and plays a role in modulating LR development [42]. Thus, we investigated whether melatonin and serotonin affected *XTR6* expression in roots. *pXTR6:GUS* seedlings were treated with 10 or 50 μM melatonin or serotonin. The expression of pXTR6:GUS was markedly stronger in melatonin- and serotonin-treated root cell layers surrounding the LRP (Fig. 2b). qRT-PCR analysis results showed that the transcription levels of the *LBD16* and *XTR6* genes were increased in melatonin- and serotonin-treated Col-0 roots, consistent with the GUS staining results (Fig. 2c and d).

Auxin plays important roles in regulating root system growth and development. The altered root system architecture (RSA) observed raised the question of whether melatonin and serotonin affect auxin accumulation and perception. Therefore, we examined auxin levels in root tips by using the auxin-responsive *DII-VENUS* transgenic marker line [43]. The DII-VENUS protein is rapidly degraded in response to auxin and can be used to visualize dynamic changes in the cellular auxin distribution. Exogenous IAA markedly decreased VENUS fluorescence in root tips, whereas neither melatonin nor serotonin showed any effect (Fig. 2e and f). Consistent with the expression pattern detected in PR tips, neither melatonin nor serotonin affected DII-VENUS expression in LRs (Fig. 2g and h). These results indicated that moderate concentrations of melatonin and serotonin did not affect auxin accumulation in roots. We then investigated whether melatonin and serotonin affected auxin transport using the *AUX1-YFP* and *PIN1/2/4/7-GFP* transgenic marker lines and found that neither melatonin nor serotonin affected the abundances of these auxin carriers (Additional file 3: Figure S3).

### Leaf physiology in response to melatonin and serotonin

**Fig. 3:**
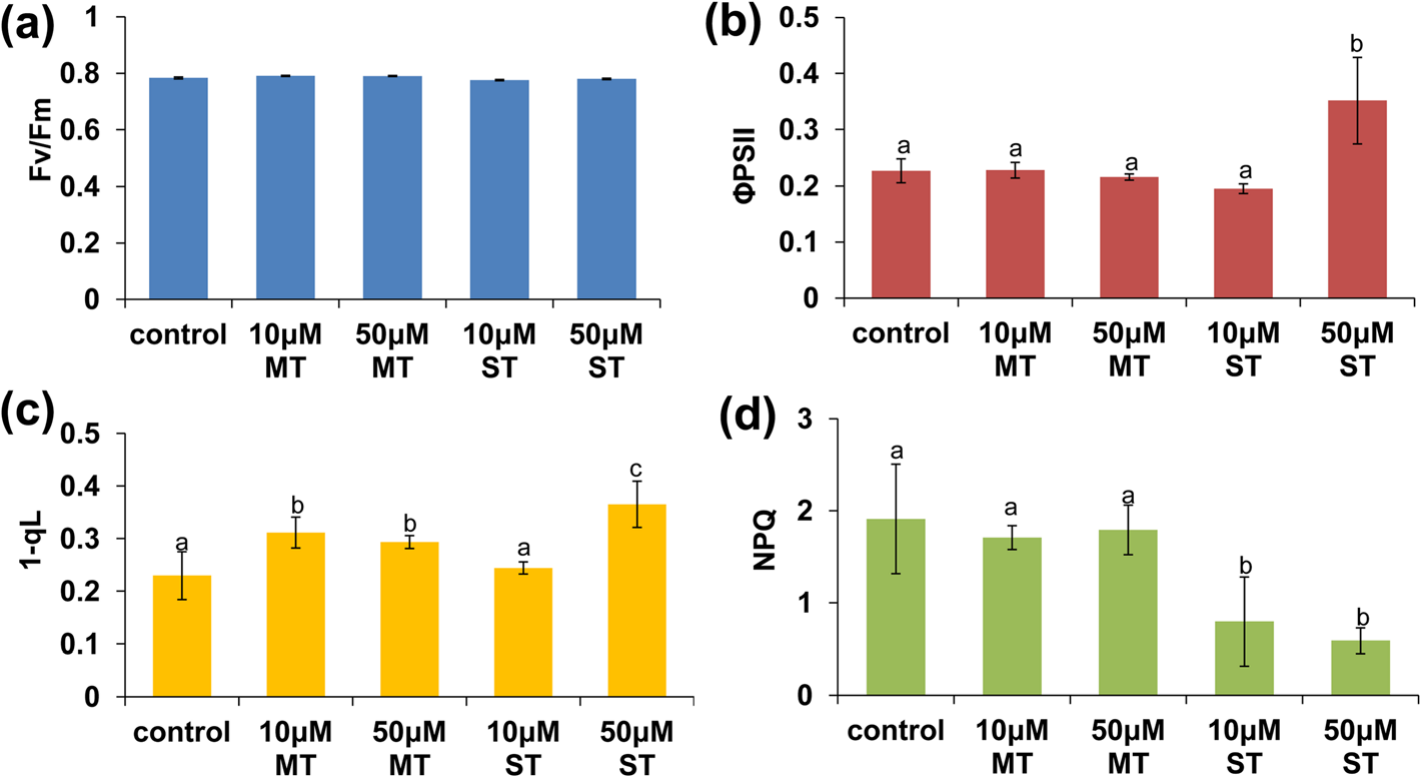
**a-d** Effects of melatonin and serotonin on photosynthesis. **a** Fv/Fm, **b** ΦPSII, **c** the PQ pool redox status, and **d** NPQ were measured in soil-grown Col-0 plants sprayed with DMSO (solvent control) or 10 or 50 μM melatonin or serotonin for 6 h. *n* = 8. Error bars represent SE. Different letters indicate significantly different values (P < 0.05 by Tukey’s test)

The above results indicated that melatonin and serotonin affect root system development. Next, we investigated the physiological consequences of melatonin and serotonin in photosynthesis. For this purpose, we measured chlorophyll fluorescence in intact leaves (Fig. 3). Compared to untreated plants, the effective quantum yield (ΦPSII) is significantly elevated in 50 μM serotonin-treated leaves, indicating a functional increase of PSII in 50 μM serotonin-treated leaves. We also monitored 1-qL, which reflects the PQ redox state of PSII, and found that it was elevated in 10 and 50 μM melatonin- and 50 μM serotonin-treated leaves. By contrast, NPQ was reduced in 10 and 50 μM serotonin-treated leaves, suggesting a lower proportion of energy dissipation in the form of heat due to the increase in PSII activity.

### Transcriptome changes in melatonin-and serotonin-treated seedlings

To elucidate the molecular mechanisms underlying melatonin- and serotonin-mediated plant growth and development, we analyzed RNA-seq data from 10 or 50 μM melatonin- or serotonin-treated seedlings with three biological replicates (Additional file 4: Supplemental Materials and methods). We sequenced 15 samples using the BGISEQ-500 platform, generating approximately 23.6 M reads per sample on average. The average mapping ratio with the reference genome was 98.35%, and the average mapping ratio with genes was 96.95%. A total of 25,160 genes were detected. We then identified differentially expressed genes (DEGs) in melatonin- and serotonin-treated seedlings based on comparison with the untreated control (log_2_ fold-change ≥2 and adjusted *P* value ≤0.05). A total of 426, 202, 426, and 259 DEGs were obtained in 10 μM melatonin-, 50 μM melatonin-, 10 μM serotonin-, and 50 μM serotonin-treated seedlings, respectively (Additional file 5: Figure S4, Additional files 6, 7, 8 and 9: Tables S1, S2, S3 and S4). All sequencing data were archived in the Short Read Archive (SRA) of the National Center for Biotechnology Information (NCBI) under accession no SRP153782.

According to the DEG results, we performed Kyoto Encyclopedia of Genes and Genomes (KEGG) pathway classification (Additional file 10: Figure S5) and functional enrichment analysis (Additional file 11: Figure S6). The DEGs were enriched in amino acid metabolism (glycine, serine, threonine, cysteine, alanine, aspartate, glutamate, methionine, and tryptophan metabolism and the biosynthesis of phenylalanine, tyrosine, and tryptophan), biosynthesis of secondary metabolites, plant-pathogen interactions, and the glutathione metabolism pathway, suggesting that supplementation with melatonin and serotonin changes both primary and secondary metabolism profiles, induces pathogen resistance, and activates ROS scavenging in plants. The DEGs in the 50 μM melatonin-treated seedlings also showed enrichment in plant hormone signal transduction, and the DEGs in 50 μM serotonin-treated seedlings showed enrichment in photosynthesis. The results were consistent with the above-described physiological data showing that supplementation with 50 μM serotonin markedly improved photosynthesis efficiency in leaves and indicated that functional differences that occurred under treatment with different concentrations of melatonin and serotonin mediated plant growth and development.

### Modulation of photosynthesis and carbon assimilation by melatonin and serotonin

**Fig. 4:**
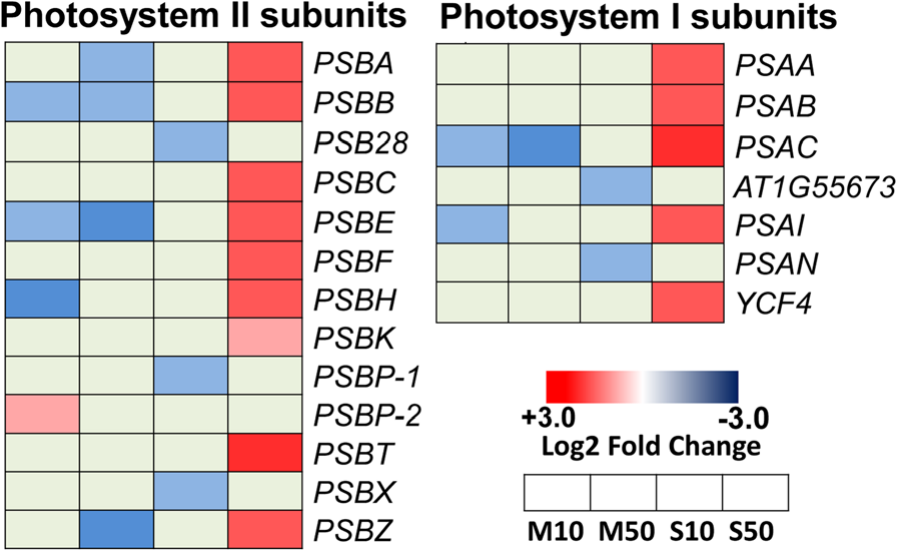
Melatonin- and serotonin-induced changes in the expression profiles of key photosynthetic genes. Gradient colors indicate the log_2_ fold-change in gene expression under different treatments compared to the untreated control. Heat maps of genes in photosystem I and photosystem II. M10, 10 μM melatonin; M50, 50 μM melatonin; S10, 10 μM serotonin; S50, 50 μM serotonin

The results described above indicated that treatment with 50 μM serotonin improves photosynthesis efficiency. Thus, we investigated genes involved in photosynthesis (Fig. 4). The expression of seven genes encoding the subunits of photosystem I (PSI) and thirteen genes encoding the subunits of photosystem II (PSII) proteins was induced in 50 μM serotonin-treated seedlings, whereas treatment with melatonin and 10 μM serotonin inhibited the expression of several genes encoding the subunits of PSI and PSII. Consistent with the elevated expression of photosynthetic process-related genes, treatment with 50 μM serotonin also markedly induced the expression of the Rubisco large subunit *RbcL* gene, a key gene in the Calvin-Benson cycle (Additional file 12: Figure S7).

### Effects of melatonin and serotonin on the carbon catabolic pathway

**Fig. 5:**
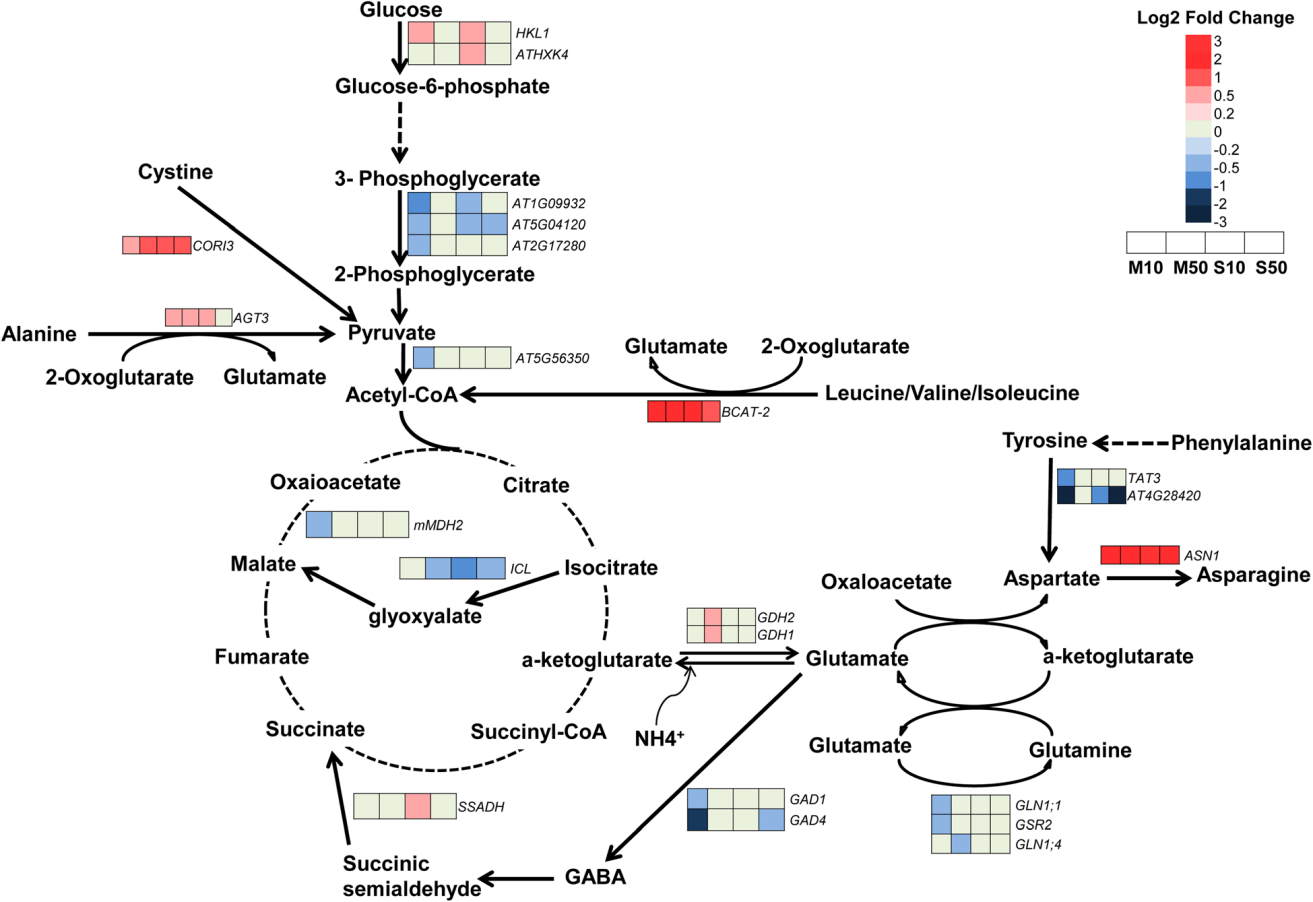
Melatonin- and serotonin-induced changes in expression profiles related to glycolysis, the TCA cycle, the glyoxylic acid cycle, primary nitrogen metabolism, and the catabolism of several key amino acids. Heat maps indicate a log_2_ fold-change in gene expression in different treatments compared to the untreated control. M10, 10 μM melatonin; M50, 50 μM melatonin; S10, 10 μM serotonin; S50, 50 μM serotonin

To further elucidate the roles of melatonin and serotonin in modulating energy metabolism, we investigated the genes involved in carbon catabolism. In the glycolysis pathway (Fig. 5), treatment with 10 μM melatonin and 10 or 50 μM serotonin repressed the expression of *phosphoglycerate mutases* (*PGMs*), and 10 μM melatonin also repressed the expression of *pyruvate kinase* (*PK*). By contrast, treatment with 10 μM melatonin and 10 μM serotonin upregulated the gene expression of *hexokinases HKL1* and *HXK4*. However, 50 μM melatonin did not affect the expression of genes encoding glycolysis enzymes.

We also analyzed genes encoding TCA cycle and glyoxylic acid cycle enzymes and found that 50 μM melatonin and 10 and 50 μM serotonin downregulated the expression of the isocitrate lyase *ICL* gene in the TCA cycle, whereas 10 μM melatonin downregulated the expression of the malate dehydrogenase (*MDH2*) gene in the glyoxylic acid cycle (Fig. 5).

### Modulation of primary nitrogen assimilation and amino acid metabolism by melatonin and serotonin

The KEGG pathway classification and functional enrichment results indicated that melatonin and serotonin affect amino acid metabolism. Therefore, we analyzed the genes involved in primary nitrogen assimilation (Fig. 5). Ammonia is primarily assimilated into glutamate via glutamate dehydrogenases or glutamate synthases [44]. The assimilation of this organic nitrogen into asparagine, a key amino acid responsible for the long-distance translocation of organic nitrogen from source to sink, then occurs via asparagine synthetase [45–47]. We found that both melatonin and serotonin induced the expression of the asparagine synthetase *ASN1*, and 50 μM melatonin also upregulated the expression of two glutamate dehydrogenases, *GDH1* and *GDH2*. These data indicated that melatonin and serotonin promote primary nitrogen assimilation.

Glutamine is an important nitrogen donor that plays a role in the synthesis of proteins and lipids and is synthesized by glutamine synthetase [48]. Melatonin downregulated the expression of three glutamine synthetases, which were not affected by serotonin, suggesting the differential functions of melatonin and serotonin in modulating nitrogen assimilation (Fig. 5).

Aminotransferases play important roles in redirecting nitrogen resources to different pathways [49]. Therefore, we analyzed the gene expression of aminotransferases (Fig. 5). The alanine aminotransferase gene *AGT3* (conversion of alanine to glutamate) and the branched-chain amino acid aminotransferase gene *BCAT-2* (conversion of leucine/valine/isoleucine to glutamate) were upregulated in melatonin- and serotonin-treated seedlings. By contrast, 10 μM melatonin repressed the expression of the tyrosine aminotransferase *TAT3*, and 10 μM melatonin and 10 or 50 μM serotonin repressed the expression of the tyrosine aminotransferase *At4G28420*.

γ-Aminobutyric acid (GABA) is a nonproteinogenic amino acid derived from glutamate that plays a vital role in modulating insect resistance in plants [48]. Treatment with 10 μM melatonin downregulated the expression of *GAD1* and *GAD4*, which are two glutamate decarboxylases that catalyze the decarboxylation of glutamate to GABA, and 50 μM serotonin also downregulated the expression of *GAD4* (Fig. 5). Additionally, treatment with 10 μM serotonin upregulated succinate-semialdehyde dehydrogenase *SSADH* expression, an enzyme that catalyzes the conversion of succinic semialdehyde to succinate.

Amino acid catabolism must be coordinated with carbohydrate metabolism and amino acid transport activity. The nodulin MtN21-like transporter family proteins *UMAMITs* play important roles in amino acid cycling between the xylem and phloem and are linked to the nutritional state of plant growth and development [50]. Melatonin and serotonin also affect the expression of several *UMAMIT* genes (Additional file 13: Figure S8). These data indicate that melatonin and serotonin affect photosynthesis and energy metabolism, thereby altering the catabolism of amino acids.

**Fig. 6:**
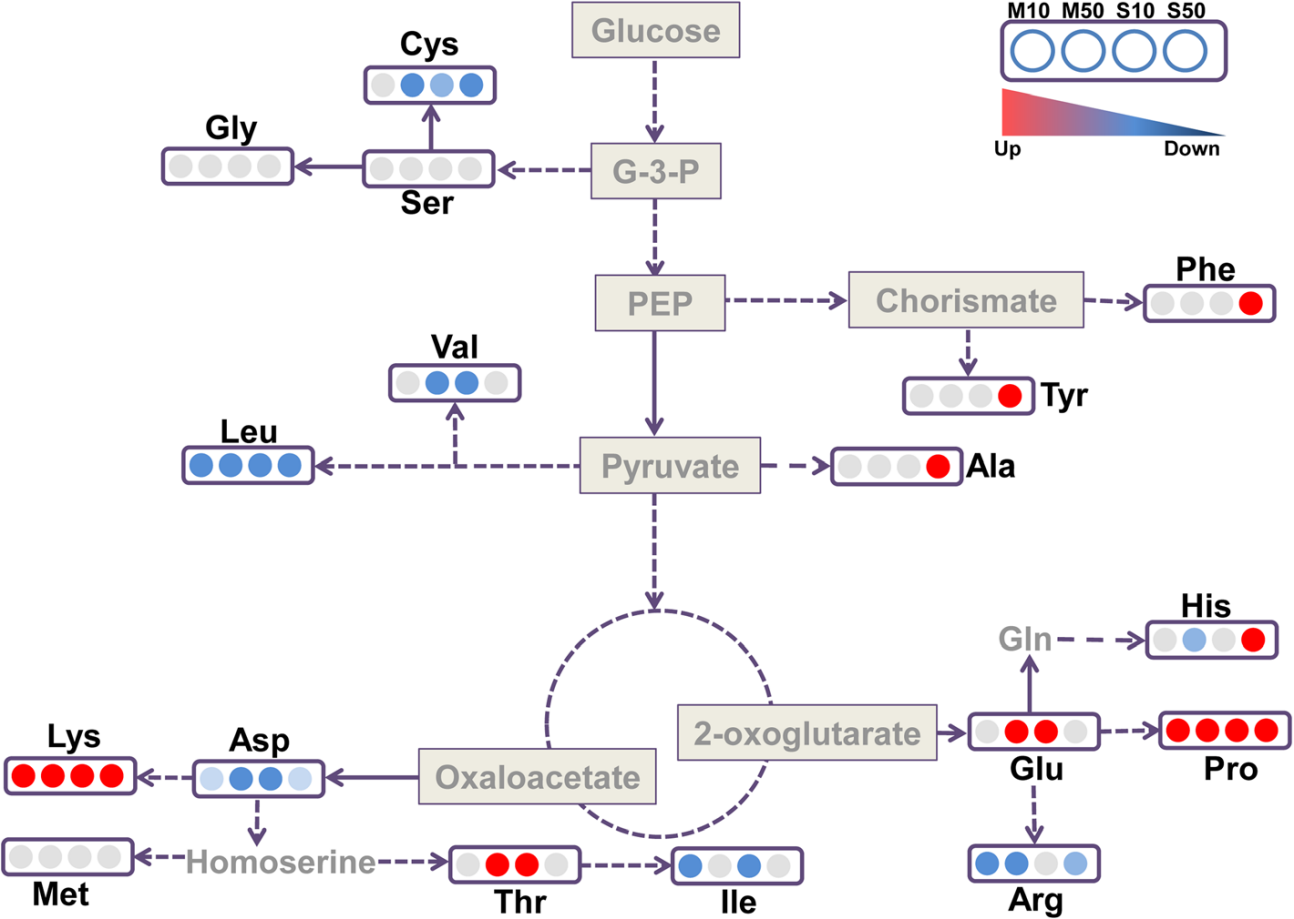
Melatonin- and serotonin-induced changes in free amino acid contents. Amino acids with significant changes in abundance compared with the untreated control are represented by red (higher) and blue (lower) circles. The gray circles indicate that the abundance of the amino acid is unaffected. These data were extracted from Table 1

We also analyzed free amino acid levels in melatonin- and serotonin-treated seedlings. Treatment with melatonin and serotonin increased the total free amino acid content in seedlings (Table 1). Both melatonin and serotonin increased the levels of Lys, Pro, Thr, and Glu in seedlings. In addition, serotonin increased the levels of Ala, Tyr, Phe, and His. By contrast, both melatonin and serotonin decreased the levels of Asp, Leu, Cys, Val, Ile, and Arg (Fig. 6, Table 1). Taken together, these data indicate that melatonin and serotonin reprogram amino acid metabolism in plants.

**Table 1:** Free amino acid content in 10 or 50 μM melatonin- and serotonin-treated seedlings

Amino acid	Abbr.	Control (mg kg^− 1^ FW)	M10 (mg kg^− 1^ FW)	M50 (mg kg^− 1^ FW)	S10 (mg kg^− 1^ FW)	S50 (mg kg^− 1^ FW)
L-aspartic acid	Asp	72.99 ± 14.7	51.2 ± 18.8*	45.69 ± 3.1*	56.5 ± 1.4*	50.1 ± 1.7*
L-threonine	Thr	95.13 ± 7.4	107.3 ± 16.1	206.2 ± 84*	218.3 ± 86.3*	114.1 ± 13.5
L-serine	Ser	30.92 ± 4.2	36 ± 5.3	42.8 ± 9.0	36.6 ± 4.8	43.7 ± 13.2
L-glutamic acid	Glu	61.6 ± 11.3	63.5 ± 13.3	106.3 ± 13.4*	125.5 ± 24.1*	70.2 ± 14.8
Glycine	Gly	98.3 ± 7.3	101.2 ± 4.8	104.8 ± 25.4	97.9 ± 14.6	105.8 ± 22.2
L-alanine	Ala	19.34 ± 6.6	18.1 ± 2.3	20.8 ± 2.2	30.2 ± 4.2*	22.9 ± 4.9
L-cysteine	Cys	38.6 ± 8.1	41.2 ± 3.4	26.9 ± 5.8*	18.7 ± 2.8*	27.8 ± 11*
L-valine	Val	73.9 ± 8.8	67.6 ± 7.7	58.3 ± 12.8*	59.8 ± 16.9*	71.1 ± 3.1
L-methionine	Met	24.7 ± 4.5	24.9 ± 3.2	25 ± 8.2	28.6 ± 1.9	33.6 ± 10.4
L-isoleucine	Ile	55.8 ± 7.0	45.1 ± 3.2*	47.8 ± 8.3	40.9 ± 6.8*	56.4 ± 9.7
L-leucine	Leu	116.9 ± 5.4	58.9 ± 1.5*	68.1 ± 17.6*	68.2 ± 12.8*	87.3 ± 6.4*
L-tyrosine	Tyr	56 ± 15.1	59.7 ± 4.9	56.8 ± 3.8	62.6 ± 21.4	91.3 ± 17*
L-phenylalanine	Phe	54.9 ± 8.8	46.5 ± 6.7	42.9 ± 7.4	44.9 ± 17.5	65.2 ± 9.2*
L-histidine	His	56.2 ± 14.3	46.1 ± 6.3	24.2 ± 6.0*	45.4 ± 20.3	82.7 ± 18*
L-lysine	Lys	70.7 ± 1.5	153.3 ± 51.9*	150.7 ± 46.9*	163.5 ± 2.3*	90.7 ± 9.8*
L-arginine	Arg	31.2 ± 2.1	24 ± 1.2*	25.9 ± 1.6*	35.8 ± 2.9	27 ± 2.2*
L-proline	Pro	11.4 ± 1.7	23.4 ± 1.3*	37.5 ± 1.6*	33.6 ± 7*	42 ± 1.6*
Total amino acid		968.7 ± 31.3	968.1 ± 23.3	1090.8 ± 45*	1167.1 ± 35.2*	1082.6 ± 86.3*

Error bars represent the SE. Asterisks indicate significant differences from the control (Student’s t test, *P* < 0.05). M10, 10 μM melatonin; M50, 50 μM melatonin; S10, 10 μM serotonin; S50, 50 μM serotonin

### Melatonin and serotonin regulate iron deficiency-responsive gene expression

**Fig. 7:**
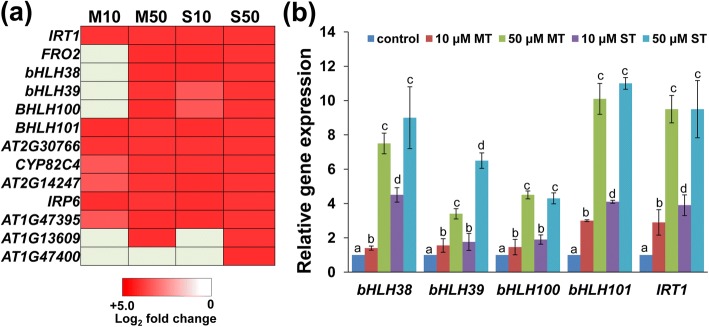
**a-b** Melatonin and serotonin induce the expression of Fe deficiency-responsive genes. **a** Heat maps indicate the log_2_ fold-change in the expression of Fe deficiency-responsive genes in different treatments compared to the untreated control. **b** Quantitative real-time reverse transcription-polymerase chain reaction (qRT-PCR) analysis of the expression of Fe uptake genes in Col-0 seedlings treated with or without 10 or 50 μM melatonin or serotonin for 1 d. The expression levels of the indicated genes in untreated roots were set to 1. Error bars represent SD. Different letters indicate significantly different values (P < 0.05 by Tukey’s test). M10, 10 μM melatonin; M50, 50 μM melatonin; S10, 10 μM serotonin; S50, 50 μM serotonin

**Fig. 8:**
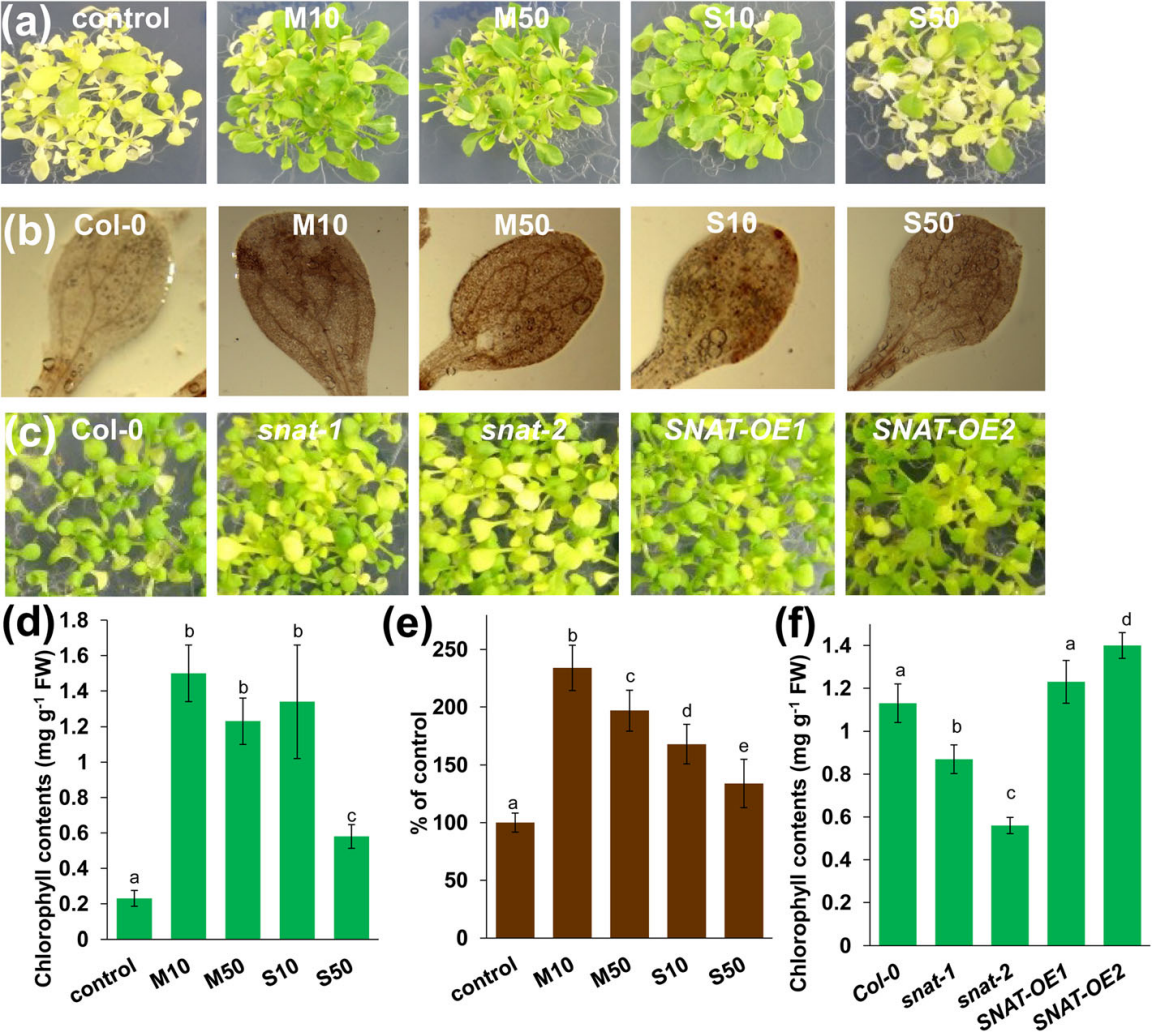
**a-f** Melatonin and serotonin improve Fe deficiency tolerance. **a, d** Phenotypic analysis (**a**) and chlorophyll contents (**d**) of seedlings grown in Fe-deficient MS agar medium without (control) or in the presence of 10 or 50 μM melatonin or serotonin for 20 d. **b, e** Images (**b**) and quantification (**e**) of Perl’s staining in seedlings grown in Fe-deficient MS agar medium with or without (control) 10 or 50 μM melatonin or serotonin for 10 d. **c**, **f** Phenotypic analysis (**c**) and chlorophyll contents (**f**) of Col-0, *snat-1*, *snat-2*, *SNAT-OE-1*, and *SNAT-OE-2* seedlings grown in Fe-deficient MS agar medium for 15 d. Error bars represent SE. Different letters indicate significantly different values (P < 0.05 by Tukey’s test). M10, 10 μM melatonin; M50, 50 μM melatonin; S10, 10 μM serotonin; S50, 50 μM serotonin

Iron (Fe) is an essential micronutrient that plays an important role in modulating plant growth and development and stress tolerance [51]. Ferric reduction oxidase 2 (FRO2) reduces Fe^3+^ to Fe^2+^, and Fe^2+^ is subsequently taken up into root cells by the iron-regulated transporter 1 (IRT1) [52]. The Fe deficiency-induced transcription factor *FIT1* interacted with the Ib subgroup of bHLH proteins (*bHLH38*, *bHLH39*, *bHLH100*, *bHLH101*) to regulate Fe acquisition in roots by inducing the expression of *FRO2* and *IRT1* [53]. Our transcriptome data revealed that both melatonin and serotonin markedly induced the expression of *bHLH38*, *bHLH39*, *bHLH100*, *bHLH101*, *FRO2*, *IRT1*, and many Fe deficiency-responsive genes under normal growth conditions (Fig. 7a), and the quantitative reverse transcription (qRT)-PCR analysis supported the above results (Fig. 7b). The qRT-PCR results were in good agreement with the RNA-seq data; this finding also verified the accuracy of the RNA-seq results. Supplementation with melatonin and serotonin alleviated Fe deficiency-induced leaf chlorosis (Fig. 8a and d). Perl staining also indicated that supplementation with melatonin or serotonin increased Fe accumulation in leaves (Fig. 8b and e).

To further confirm our results, we also analyzed the phenotypes of the melatonin biosynthesis-defective *snat* knockout mutants *snat-1* (SALK_032239) and *snat-2* (SALK_020577) [15, 54] and two *SNAT*-overexpressing *SNAT-OE-1* and *SNAT-OE-2* transgenic lines (Additional file 14: Figure S9). The loss-of-function *snat* mutants showed sensitivity to Fe deficiency, whereas the *SNAT-OE* lines showed greater tolerance to Fe deficiency (Fig. 8c and f).

## Discussion

Both melatonin and serotonin modulate RSA. Moderate levels of melatonin and serotonin do not affect PR growth, whereas high concentrations of melatonin and serotonin inhibit PR growth by repressing auxin biosynthesis, transport, and accumulation in roots [19, 40]. High concentrations of serotonin also markedly inhibit LR formation. In this study, we found that moderate concentrations of melatonin and serotonin exhibited similar functions in modulating PR growth and LR formation. Although serotonin is the precursor of melatonin, a number of studies have indicated that increased serotonin levels do not induce melatonin accumulation in plants [22–24], suggesting that the phenotype caused by exogenous serotonin in plant growth and development is not due to its conversion to melatonin [23, 24].

Treatment with melatonin and serotonin significantly increased the number of LRPs in stage I and stage IV. The increased number of LRPs in stage I indicated that melatonin and serotonin induce LR formation by increasing LRP initiation. We found that the number of LRPs in stage II and III was unaffected, whereas the number of LRPs in stage IV was markedly greater than in the control roots. These data suggested that melatonin and serotonin play greater roles in LR elongation than initiation, and rapid elongation of LRPs led to an insignificant phenotype in stages II and III. Taken together, these data indicate that melatonin and serotonin induce LR formation by increasing LRP initiation and LR elongation.

*LBD16* and *XTR6* are two important marker genes that play roles in LR emergence and elongation [41, 42]. Exogenous melatonin and serotonin markedly induced the expression of the *LBD16* and *XTR6* genes, indicating that melatonin and serotonin caused localized induction of cell-wall-remodeling-related genes, thereby inducing LR development.

The interaction between auxin and melatonin/serotonin has been a controversial issue for a number of years. Melatonin and serotonin are derived from the common substrate L-tryptophan in association with auxin. Early reports revealed that high concentrations of melatonin and serotonin decrease auxin accumulation in roots and thereby repress PR growth. Pelagio-Flores et al. (2011) showed that high concentrations of serotonin (450 μM) inhibit auxin accumulation in leaves and roots [19]. Wang et al. (2016) revealed that high concentrations of melatonin (> 100 μM) repress auxin biosynthesis and auxin transport by decreasing the abundance of auxin efflux carriers PIN1/4/7 in root tips [40]. The results of these studies suggest that melatonin and serotonin can act as either auxin inhibitors or antagonists. However, Pelagio-Flores et al. (2011, 2012) found that even treatment with high concentration of melatonin and serotonin (450 μM) did not affect auxin perception [19, 39], suggesting that the mechanism whereby melatonin regulates RSA is likely independent of auxin signaling. In this study, we found that moderate concentrations of melatonin and serotonin did not affect auxin accumulation in roots. Unlike the high-concentration treatments, moderate concentrations of melatonin and serotonin did not affect the abundance of the auxin carriers AUXI and PIN1/2/4/7. Because no changes in auxin accumulation occurred in roots, supplementation with melatonin and serotonin did not affect stem cell niche activity and meristem cell division; thus, PR growth was unaffected.

Senescence induces serotonin overaccumulation in rice leaves. Suppression of serotonin accumulation occurs by repressing the expression of the *tryptophan decarboxylase* (*TDC*) gene, which accelerates leaf senescence in rice [1], indicating that serotonin delays the senescence of rice leaves. However, the underlying molecular mechanisms remain largely unclear. In this study, we found that 50 μM serotonin significantly improved PSII activity, and the transcriptome data supported this result. Treatment with 50 μM serotonin also markedly induced the expression of *RbcL*, a key enzyme in the Calvin-Benson cycle. These data indicated that 50 μM serotonin improved photosynthesis efficiency and energy generation in plants, thereby delaying leaf senescence. However, we found that 10 or 50 μM melatonin and 10 μM serotonin slightly downregulated the expression of several photosynthesis- and Calvin-Benson cycle-related genes (log_2_ FC < 1), although the physiological analysis indicated that these treatments did not affect photosynthesis efficiency. Further analysis indicated that 50 μM melatonin did not affect the expression of genes encoding glycolysis and TCA cycle enzymes. Only one gene in the TCA cycle (*mMDH2*) was repressed by 10 μM melatonin; one gene in the glyoxylic acid cycle (*ICL*) was repressed by 50 μM melatonin and 10 or 50 μM serotonin; three phosphoglycerate mutase (*PGMs*) genes involved in glycolysis were repressed by 10 μM melatonin and 10 and 50 μM serotonin; and one pyruvate kinase gene (*PK*) was repressed by 10 μM melatonin. However, hexokinase genes (*HKL1* and *HXK4*) were induced in 10 μM melatonin- and 10 μM serotonin-treated seedlings. These results suggest that melatonin and serotonin affect glycolysis and the TCA cycle only slightly.

Melatonin reprograms carbohydrate metabolism by inducing the synthesis of carbohydrates from nonsugar precursors during sugar starvation in plants [18]. The KEGG functional enrichment results indicated that melatonin and serotonin affect amino acid metabolism. Therefore, we predicted that melatonin and serotonin affect amino acid catabolism. Indeed, we found that melatonin and serotonin regulate the distribution of nitrogen resources and amino acid breakdown to the production of sugars. Several findings supported this conclusion. First, although 50 μM melatonin did not affect the expression of genes encoding glycolysis and TCA cycle enzymes, it promoted ammonia assimilation by inducing the expression of glutamate dehydrogenases *GDH1* and *GDH2* and the asparagine synthetase *ASN1*. Second, melatonin and serotonin promoted the catabolism of leucine, valine, and isoleucine to form acetyl-CoA and that of alanine and cystine to form pyruvate, by inducing the expression of the branched-chain amino acid aminotransferase gene *BCAT-2*, the alanine aminotransferase gene *AGT3*, and the cystine lyase *CORI3*. Third, the analysis of free amino acid contents indicated that the levels of Leu, Val, Ile, and Cys were reduced in melatonin- and serotonin-treated seedlings. Taken together, these data indicate that melatonin and serotonin reprogram primary nitrogen assimilation and amino acid catabolism, thereby affecting carbon metabolism and energy metabolism in plants.

In this study, we found that both melatonin and serotonin markedly induced the expression of many Fe deficiency-responsive genes under normal growth conditions. Further physiological analysis indicated that exogenous melatonin and serotonin alleviate Fe deficiency-induced leaf chlorosis and Fe accumulation in leaves, as indicated by Perl staining. Genetics analysis using loss-of-function mutants and overexpressors also supported these results. These findings indicate that melatonin and serotonin improve tolerance to Fe deficiency in plants.

## Conclusion

In this study, changes in nitrogen metabolism and carbon metabolism were observed, which could be explained by reprogramming of the gene expression pattern. Moderate concentrations of melatonin and serotonin did not affect PR growth but markedly induced LR formation. High concentrations of melatonin and serotonin inhibited auxin accumulation and transport [2, 40], whereas moderate concentrations had no such effect, implying that melatonin- and serotonin-mediated LR formation is auxin independent. Both melatonin and serotonin induce LR formation by locally inducing the expression of cell-wall-remodeling-related genes. Melatonin and serotonin also improve photosynthesis and Fe deficiency tolerance. Further studies exploring the interplay of melatonin and serotonin with metabolism will confer a broader understanding of the mechanisms by which plant growth and development respond to melatonin and serotonin, which will be helpful for the development of cost-effective strategies for agricultural production and will also provide insight into applications for these phytoneurotransmitters.

## Additional files

Additional file 1: **Figure S1.** Effects of melatonin and serotonin on PR growth. (DOCX 401 kb)

Additional file 2: **Figure S2.** Effects of melatonin and serotonin on meristem cell division potential and stem cell niche activity. (DOCX 1287 kb)

Additional file 3: **Figure S3.** Effects of melatonin and serotonin on the abundances of auxin carriers. (DOCX 991 kb)

Additional file 4: Supplemental Materials and methods. (DOCX 12 kb)

Additional file 5: **Figure S4.** Scatter plot of DEGs. (DOCX 209 kb)

Additional file 6: **Table S1.** 10 μM melatonin-regulated genes. (XLSX 824 kb)

Additional file 7: **Table S2.** 50 μM melatonin-regulated genes. (XLSX 454 kb)

Additional file 8: **Table S3.** 10 μM serotonin-regulated genes. (XLSX 1047 kb)

Additional file 9: **Table S4.** 50 μM serotonin-regulated genes. (XLSX 383 kb)

Additional file 10: **Figure S5.** Pathway classification of DEGs. (DOCX 698 kb)

Additional file 11: **Figure S6.** Pathway functional enrichment of DEGs. (DOCX 1093 kb)

Additional file 12: **Figure S7.** Heat maps of genes involved in Calvin-Benson cycle. (DOCX 138 kb)

Additional file 13: **Figure S8.** Heat maps indicate log_2_ fold-change in the expression of *UMAMIT* genes in different treatments compared to the untreated control. (DOCX 90 kb)

Additional file 14: **Figure S9.** Expression analysis of *SNAT-OE* transgenic lines. (DOCX 87 kb)

## Abbreviations

LR: Lateral root
MT: Melatonin
PR: Primary root
ST: Serotonin
