# Supplementary material for: Comparative physiological responses and transcriptome analysis reveal the roles of melatonin and serotonin in regulating growth and metabolism in Arabidopsis
Source: BMC Plant Biol. 2018 Dec 18;18:362. doi: 10.1186/s12870-018-1548-2 (PMC6299670; doi:10.1186/s12870-018-1548-2)
Supplement: Supplementary file 1 — Figure S1. Effects of melatonin and serotonin on PR growth. (DOCX 401 kb) [file 12870_2018_1548_MOESM1_ESM.docx]

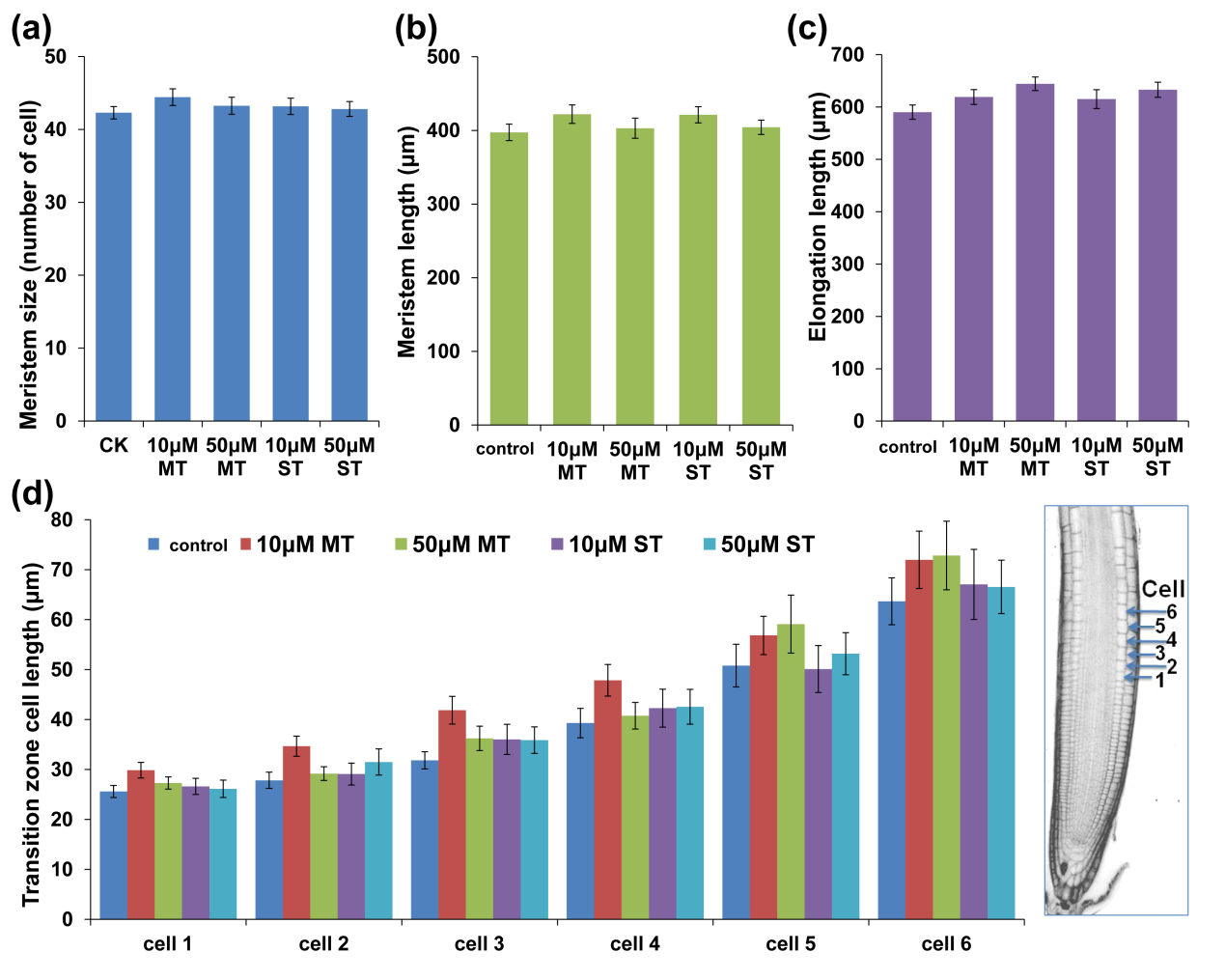


**Figure S1.** Effects of melatonin and serotonin on PR growth. Five-day-old seedlings were transferred to 1/4 MS medium containing 10 or 50 μM melatonin or serotonin for 4 days. (A) Meristem size (number of cells), (B) meristem length, (C) elongation length, and (D) transition zone cell length were determined. MT, melatonin; ST, serotonin. Error bars represent the SE.
