# Supplementary material for: Comparative physiological responses and transcriptome analysis reveal the roles of melatonin and serotonin in regulating growth and metabolism in Arabidopsis
Source: BMC Plant Biol. 2018 Dec 18;18:362. doi: 10.1186/s12870-018-1548-2 (PMC6299670; doi:10.1186/s12870-018-1548-2)
Supplement: Supplementary file 2 — Figure S2. Effects of melatonin and serotonin on meristem cell division potential and stem cell niche activity. (DOCX 1287 kb) [file 12870_2018_1548_MOESM2_ESM.docx]

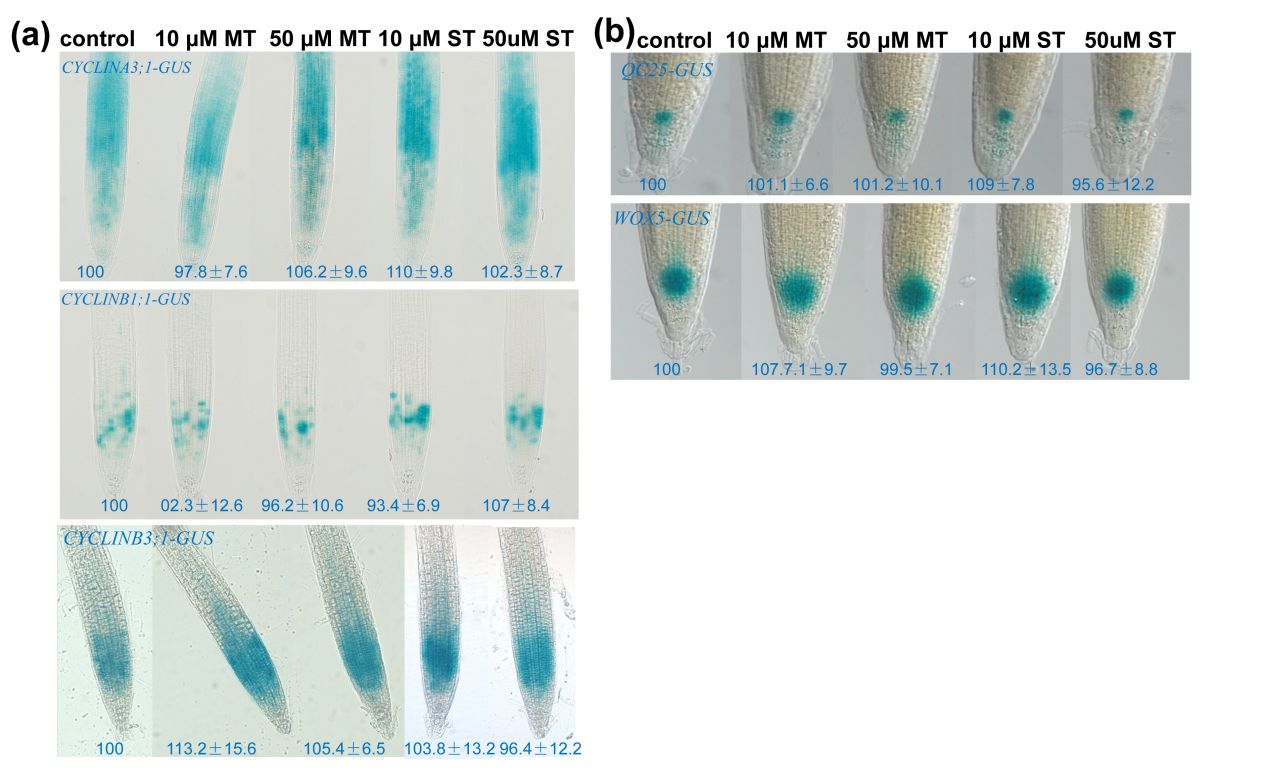


**Figure S2.** Effects of melatonin and serotonin on (A) meristem cell division potential and (B) stem cell niche activity. Images of GUS staining of 5-day-old (A) *CYCLINA3;1-GUS*, *CYCLINB1;1-GUS*, and *CYCLINB3;1-GUS* and (B) QC25-GUS and WOX5-GUS seedlings exposed to 10 or 50 μM melatonin or serotonin for 4 days. MT, melatonin; ST, serotonin. The numbers represent the relative GUS activity (% of control). The GUS activity in the untreated roots (control) was set to 100.
