# Supplementary material for: Comparative physiological responses and transcriptome analysis reveal the roles of melatonin and serotonin in regulating growth and metabolism in Arabidopsis
Source: BMC Plant Biol. 2018 Dec 18;18:362. doi: 10.1186/s12870-018-1548-2 (PMC6299670; doi:10.1186/s12870-018-1548-2)
Supplement: Supplementary file 3 — Figure S3. Effects of melatonin and serotonin on the abundances of auxin carriers. (DOCX 991 kb) [file 12870_2018_1548_MOESM3_ESM.docx]

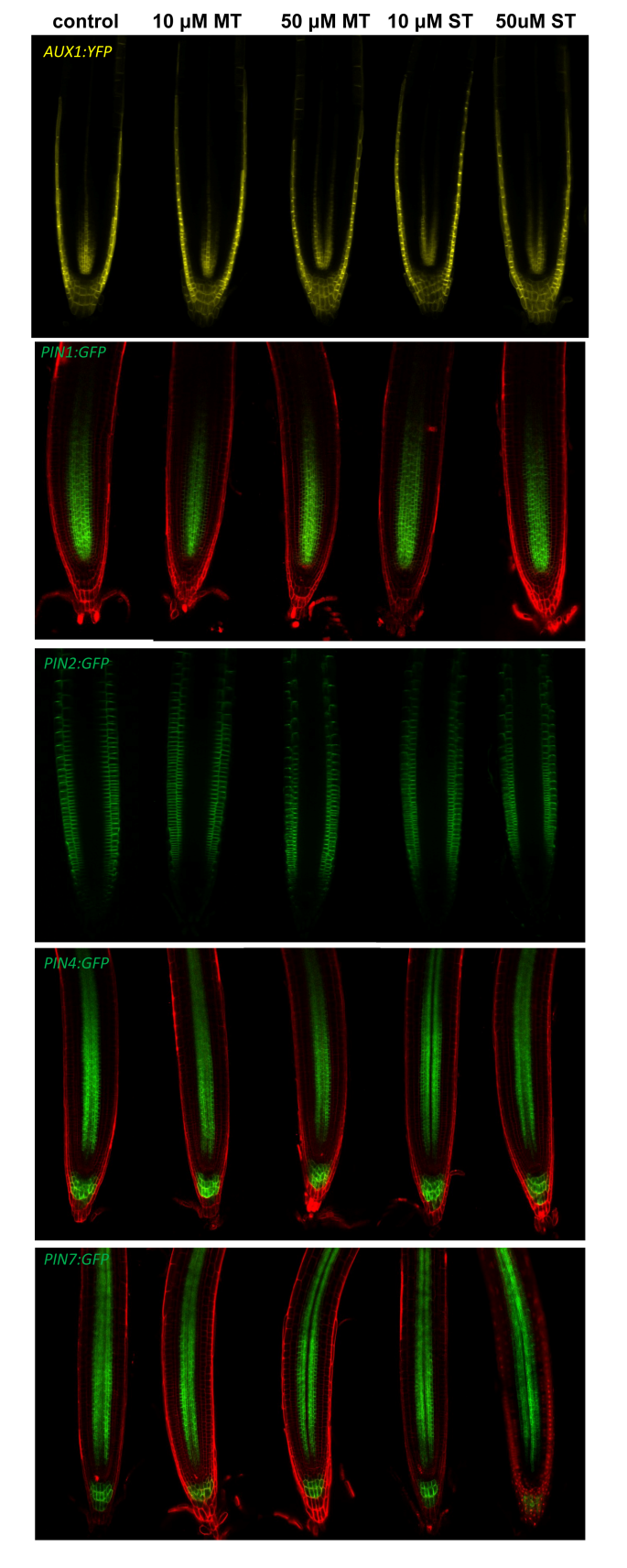


**Figure S3.** Effects of melatonin and serotonin on the abundances of auxin carriers. GFP/YFP fluorescence in the roots of 5-day-old *AUX1:YFP*, *PIN1:GFP*, *PIN2:GFP*, *PIN4:GFP*, and *PIN7:GFP* seedlings exposed to 10 or 50 μM melatonin or serotonin for 4 days.
