# Supplementary material for: Comparative physiological responses and transcriptome analysis reveal the roles of melatonin and serotonin in regulating growth and metabolism in Arabidopsis
Source: BMC Plant Biol. 2018 Dec 18;18:362. doi: 10.1186/s12870-018-1548-2 (PMC6299670; doi:10.1186/s12870-018-1548-2)
Supplement: Supplementary file 4 — Supplemental Materials and methods. (DOCX 12 kb) [file 12870_2018_1548_MOESM4_ESM.docx]

**Supplemental Materials and Methods**

*Digital transcriptomics*

Total RNAs were extracted from 5-d-old *Arabidopsis* Col-0 seedlings treated with or without 10 μM or 50 μM serotonin or melatonin for 2 d using RNAiso Plus (TaKaRa) according to the manufacturer’s instructions. The cDNAs were synthesized using the PrimeScript^TM^ RT Reagent Kit with gDNA Eraser (TaKaRa). Oligo (dT) magnetic bead adsorption was used to purify the mRNA, and the mRNA was randomly fragmented using fragmentation buffer. The 6-bp-random hexamers were used as a primer to synthesize the first and second strand cDNA. AMPure XP beads were used to purify the cDNA. After end repair and adding A tail, the purified double-strand cDNA was linked with the sequencing adaptor and then subjected to fragment size selection using AMPure XP beads. The cDNA library was obtained by PCR amplification. The concentration of the cDNA library was analyzed using Qubit 2.0 and Agilent 2100.

High-throughput sequencing was performed using BGISEQ-500 platform, averagely generating 23.6 M reads per samples. The raw sequences were transformed into clean tags after certain steps of data processing were performed, including the removal of the adaptor sequence, empty reads, and low-quality tags; finally, clean tags were generated. Sequences from Arabidopsis (TAIR 10) were used to obtain the reference gene sequence. All of the clean tags were mapped to the reference sequences. The clean tags that mapped to the reference sequences of multiple genes were filtered. The average mapping ratio with reference genome is 98.35%, the average mapping ratio with gene is 96.95%; A total of 25,160 genes were detected.

The differentially expressed genes were detected using DEGseq2 algorithms (Michael et al., 2014). The P value corresponds to the differential gene expression test. We used the log2Ratio > 0.5 and the adjusted p-value ≤ 0.05 as the threshold to judge the differential expressed genes. High-throughput sequencing was performed with the assistance of BGI Corporation (Wuhan, China).

**Supplemental References**

Love MI, Huber W, Anders S. Moderated estimation of fold change and dispersion for RNA-seq data with DESeq2. Genome Biol. 2014;15:550.
