# Supplementary material for: Comparative physiological responses and transcriptome analysis reveal the roles of melatonin and serotonin in regulating growth and metabolism in Arabidopsis
Source: BMC Plant Biol. 2018 Dec 18;18:362. doi: 10.1186/s12870-018-1548-2 (PMC6299670; doi:10.1186/s12870-018-1548-2)
Supplement: Supplementary file 5 — Figure S4. Scatter plot of DEGs. (DOCX 209 kb) [file 12870_2018_1548_MOESM5_ESM.docx]

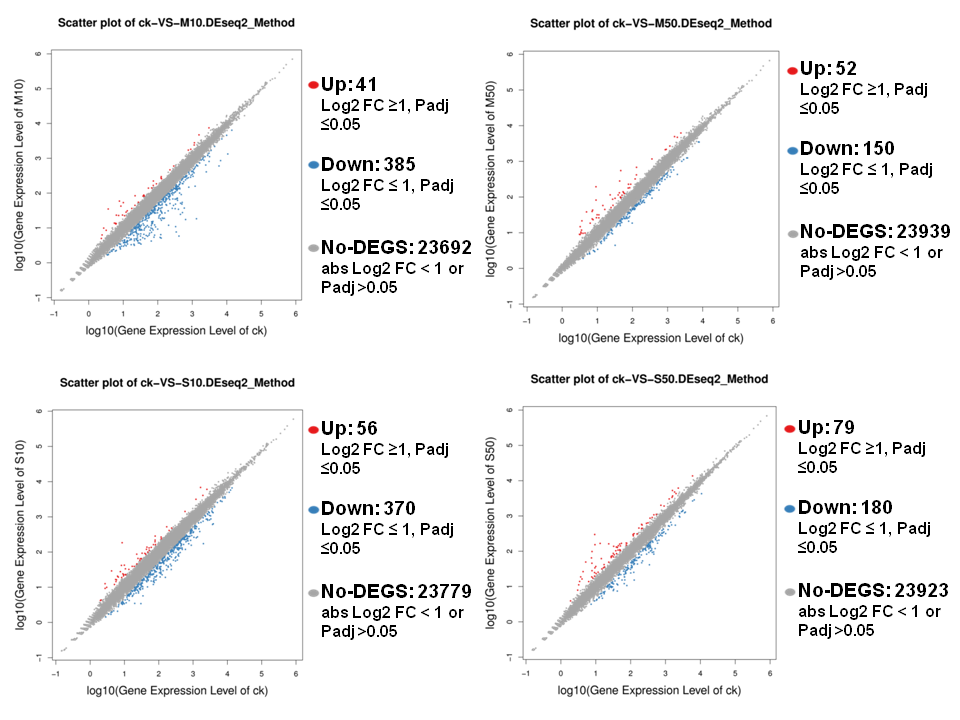


**Figure S4.** Scatter plot of differential expressed genes (DEGs). X and Y axis represent log10 transformed gene expression level, red color represents the up-regulated genes, blue color represents the down-regulated genes, gray color represents the non-DEGs. M10, 10 μM melatonin; M50, 50 μM melatonin; S10, 10 μM serotonin; S50, 50 μM serotonin.
