# Supplementary material for: Comparative physiological responses and transcriptome analysis reveal the roles of melatonin and serotonin in regulating growth and metabolism in Arabidopsis
Source: BMC Plant Biol. 2018 Dec 18;18:362. doi: 10.1186/s12870-018-1548-2 (PMC6299670; doi:10.1186/s12870-018-1548-2)
Supplement: Supplementary file 10 — Figure S5. Pathway classification of DEGs. (DOCX 698 kb) [file 12870_2018_1548_MOESM10_ESM.docx]

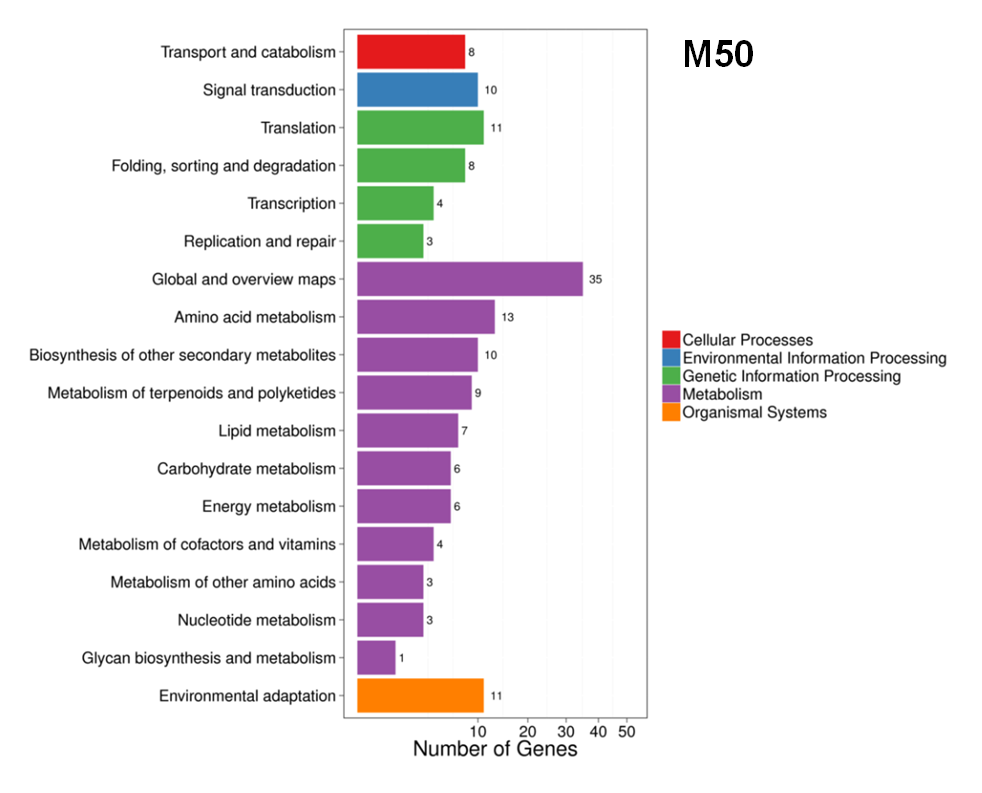

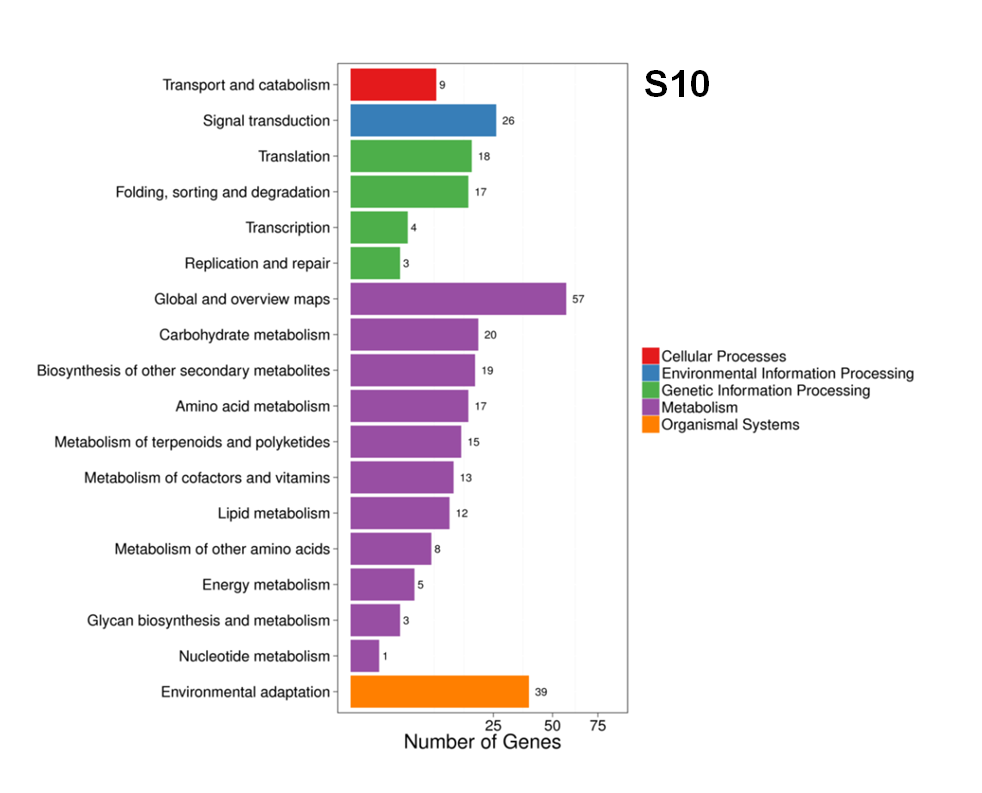

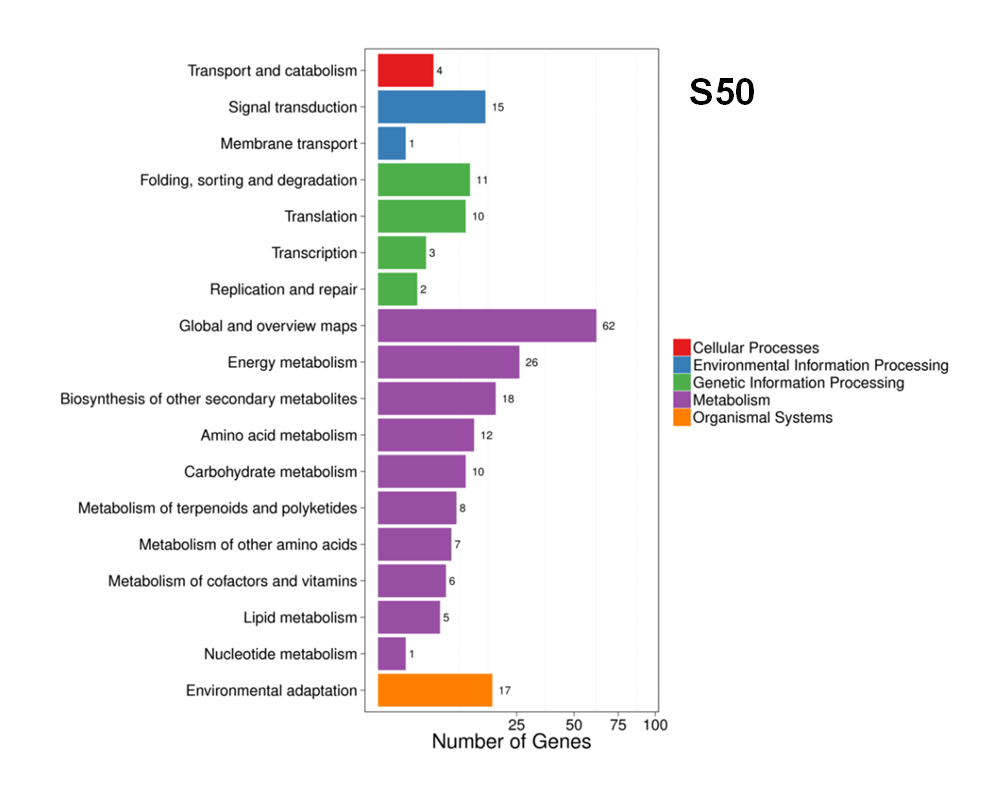


**Figure S5.** Pathway classification of differential expressed genes (DEGs). X axis represents number of DEG. Y axis represents functional classification of KEGG. There are seven branches for KEGG pathways: Cellular Processes, Environmental Information Processing, Genetic Information Processing, Metabolism, and Organismal Systems. M10, 10 μM melatonin; M50, 50 μM melatonin; S10, 10 μM serotonin; S50, 50 μM serotonin.
