# Supplementary material for: Comparative physiological responses and transcriptome analysis reveal the roles of melatonin and serotonin in regulating growth and metabolism in Arabidopsis
Source: BMC Plant Biol. 2018 Dec 18;18:362. doi: 10.1186/s12870-018-1548-2 (PMC6299670; doi:10.1186/s12870-018-1548-2)
Supplement: Supplementary file 11 — Figure S6. Pathway functional enrichment of DEGs. (DOCX 1093 kb) [file 12870_2018_1548_MOESM11_ESM.docx]

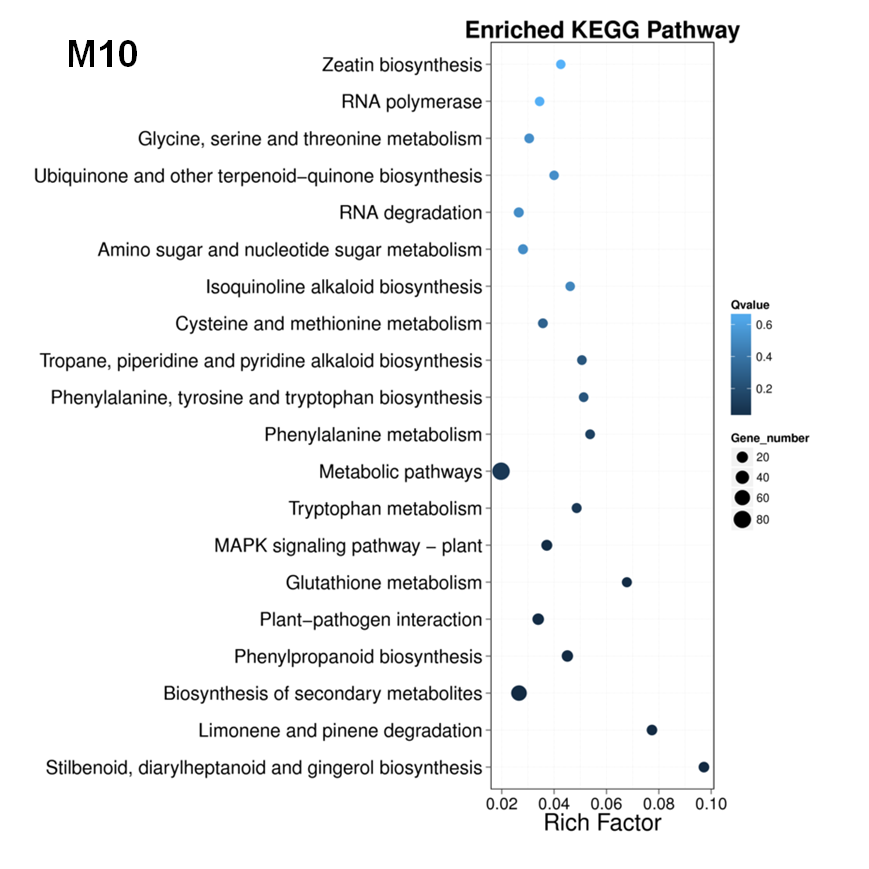


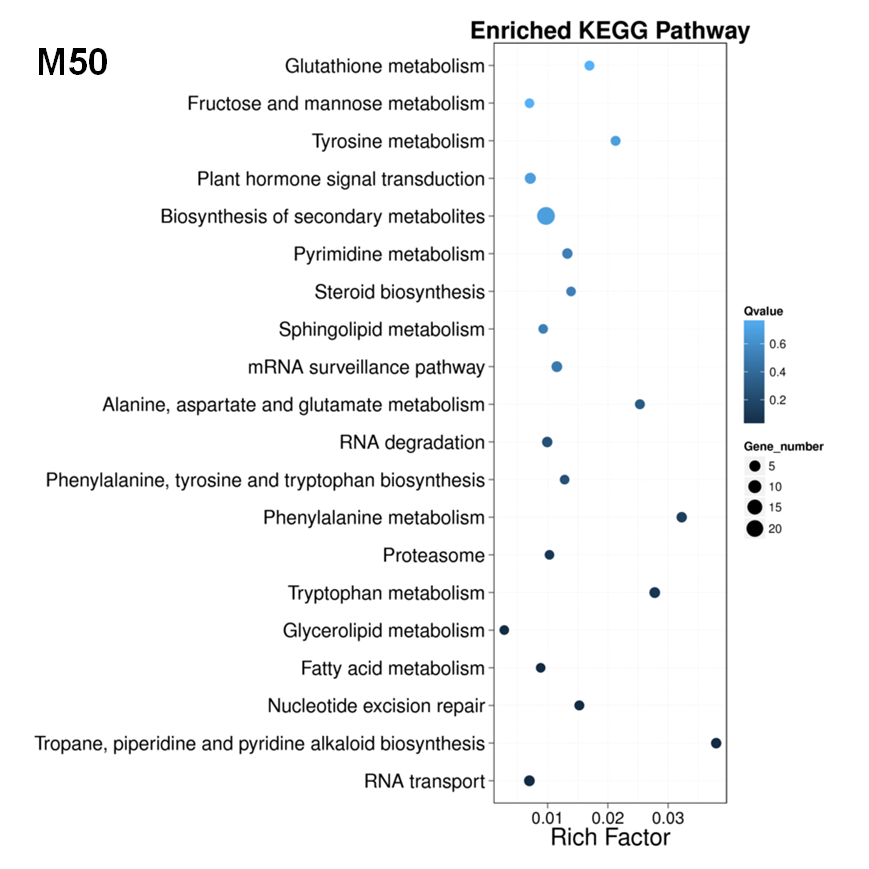

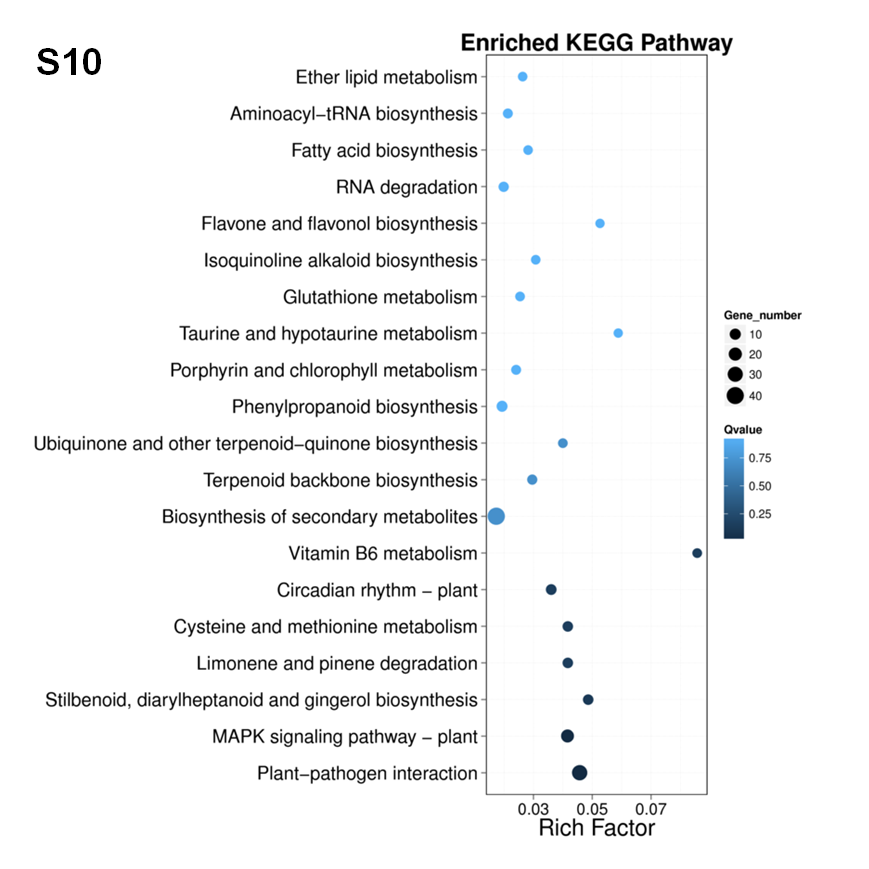


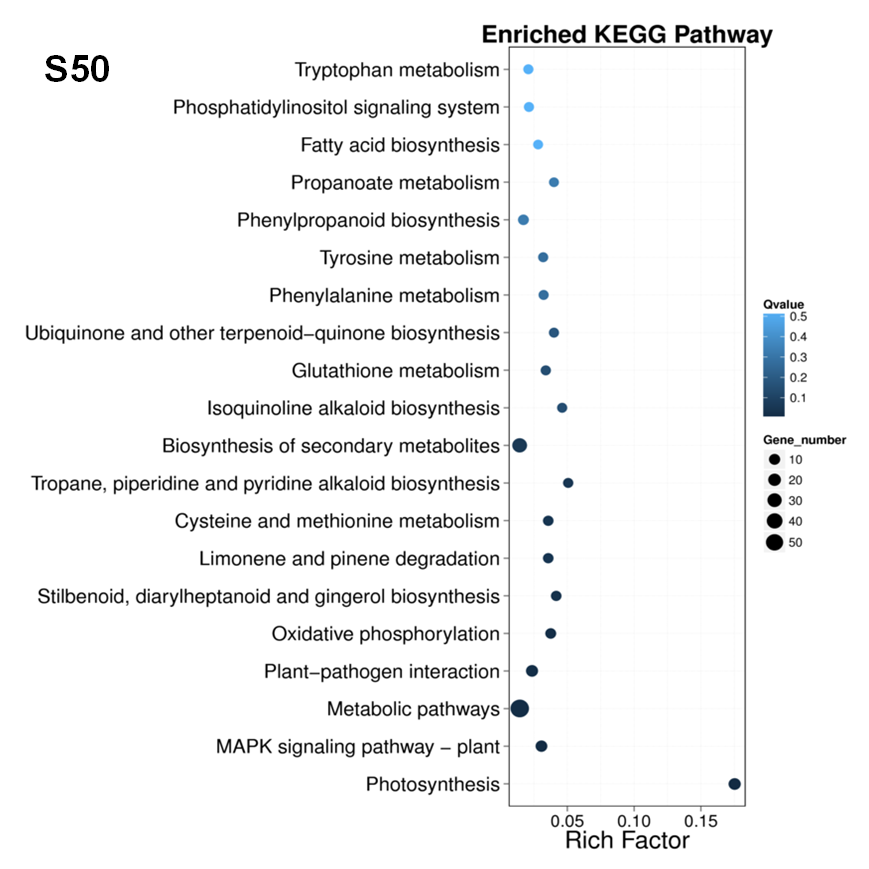


**Figure S6.** Pathway functional enrichment of differential expressed genes (DEGs). X axis represents enrichment factor. Y axis represents pathway name. The color indicates the q-value (high: white, low: blue), the lower q-value indicates the more significant enrichment. Point size indicates DEG number (The bigger dots refer to larger amount). Rich Factor refers to the value of enrichment factor, which is the quotient of foreground value (the number of DEGs) and background value (total Gene amount). The larger the value, the more significant enrichment. M10, 10 μM melatonin; M50, 50 μM melatonin; S10, 10 μM serotonin; S50, 50 μM serotonin.
