# Supplementary material for: Comparative physiological responses and transcriptome analysis reveal the roles of melatonin and serotonin in regulating growth and metabolism in Arabidopsis
Source: BMC Plant Biol. 2018 Dec 18;18:362. doi: 10.1186/s12870-018-1548-2 (PMC6299670; doi:10.1186/s12870-018-1548-2)
Supplement: Supplementary file 12 — Figure S7. Heat maps of genes involved in Calvin-Benson cycle. (DOCX 138 kb) [file 12870_2018_1548_MOESM12_ESM.docx]

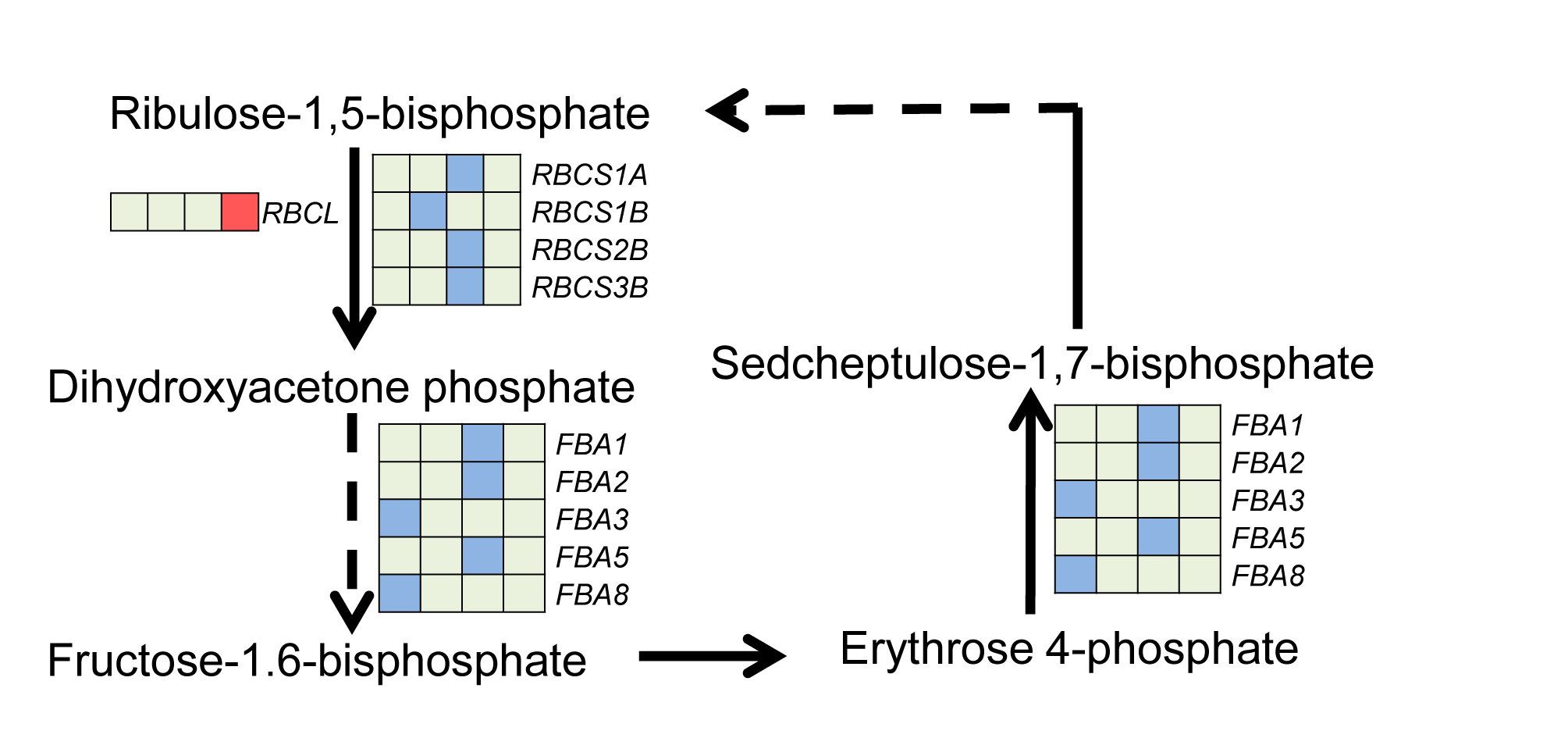


**Figure S7.** Heat maps of genes involved in Calvin-Benson cycle. M10, 10 μM melatonin; M50, 50 μM melatonin; S10, 10 μM serotonin; S50, 50 μM serotonin.
