# Supplementary material for: Comparative physiological responses and transcriptome analysis reveal the roles of melatonin and serotonin in regulating growth and metabolism in Arabidopsis
Source: BMC Plant Biol. 2018 Dec 18;18:362. doi: 10.1186/s12870-018-1548-2 (PMC6299670; doi:10.1186/s12870-018-1548-2)
Supplement: Supplementary file 13 — Figure S8. Heat maps indicate log2 fold-change in the expression of UMAMIT genes in different treatments compared to the untreated control. (DOCX 90 kb) [file 12870_2018_1548_MOESM13_ESM.docx]

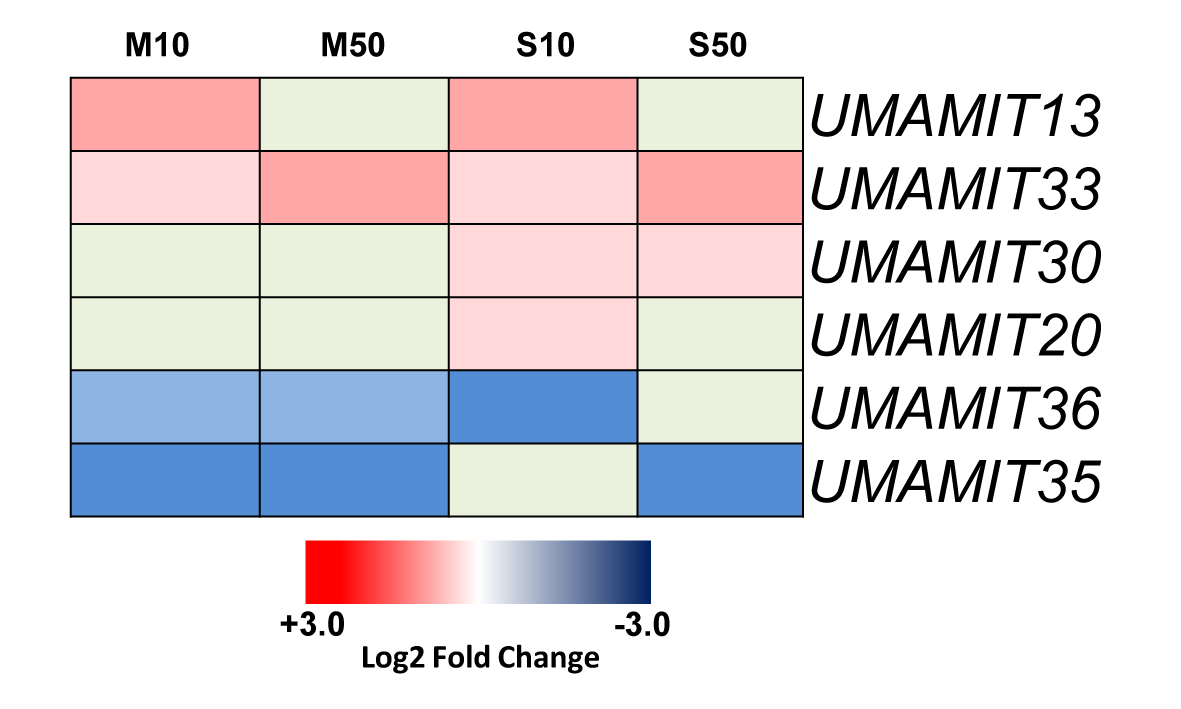


**Figure S8.** Heat maps indicate log_2_ fold-change in the expression of *UMAMIT* genes in different treatments compared to the untreated control. M10, 10 μM melatonin; M50, 50 μM melatonin; S10, 10 μM serotonin; S50, 50 μM serotonin.
