# Supplementary material for: Comparative physiological responses and transcriptome analysis reveal the roles of melatonin and serotonin in regulating growth and metabolism in Arabidopsis
Source: BMC Plant Biol. 2018 Dec 18;18:362. doi: 10.1186/s12870-018-1548-2 (PMC6299670; doi:10.1186/s12870-018-1548-2)
Supplement: Supplementary file 14 — Figure S9. Expression analysis of SNAT-OE transgenic lines. (DOCX 87 kb) [file 12870_2018_1548_MOESM14_ESM.docx]

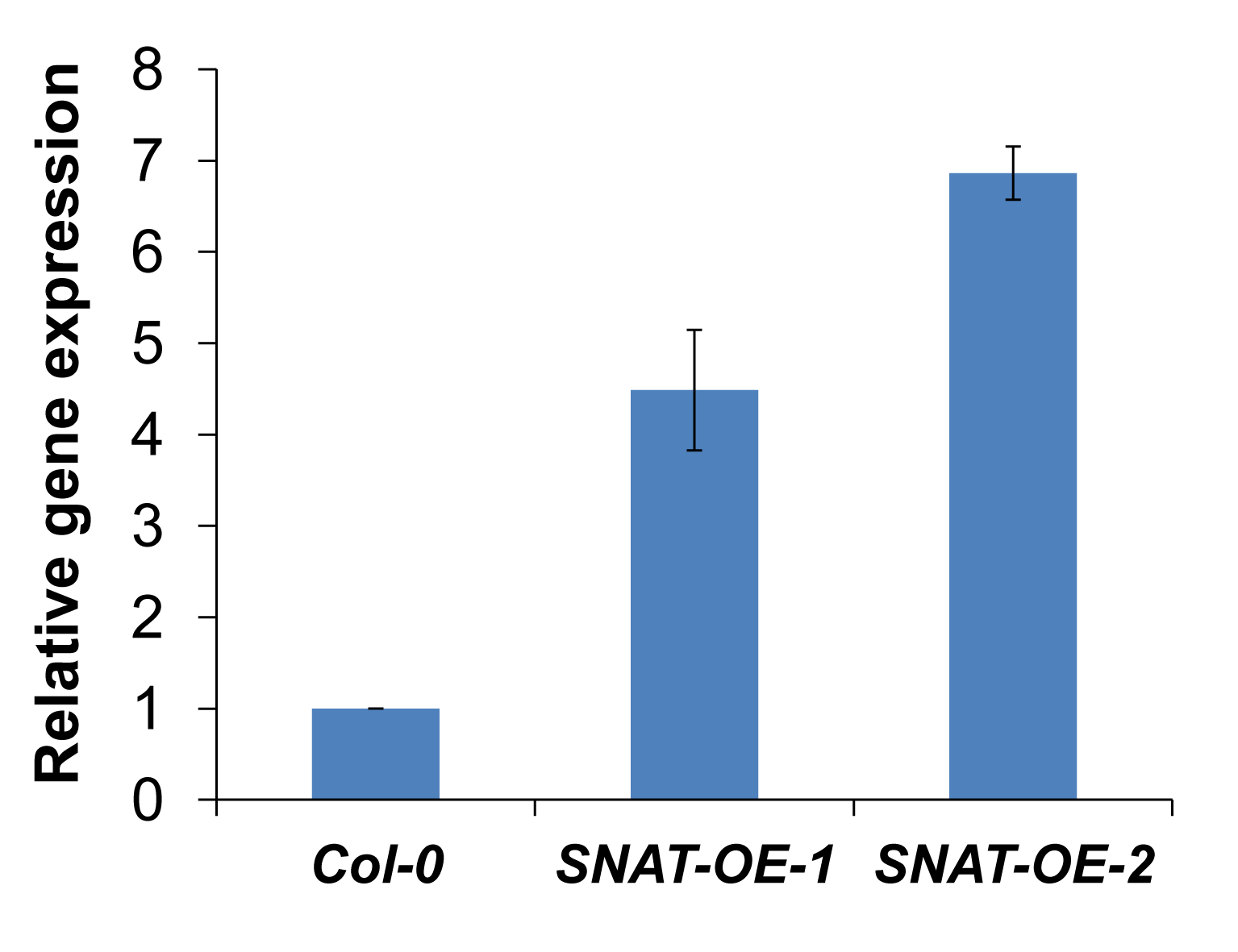


**Figure S9.** qRT-PCR analysis of *AtSNAT* mRNA levels in Col-0 and two overexpressed transgenic lines *SNAT-OE-1* and *SNAT-OE-2*.
